# Silica-Embedded Proteins Enable Higher Yield and Atomic Ion Detection in Deep-UV Laser-Assisted Atom Probe Tomography

**DOI:** 10.1021/acs.analchem.6c02770

**Published:** 2026-08-14

**Authors:** Gustav Eriksson, David Mayweg, Giovanni Novi Inverardi, Francesco Carnovale, Mats Hulander, Mattias Thuvander, Lorenzo Petrolli, Gianluca Lattanzi, Simone Taioli, Martin Andersson

**Affiliations:** † 211731 Chalmers University of Technology, Department of Chemistry and Chemical Engineering, Kemigården 4, 41296 Gothenburg, Sweden; ‡ Chalmers University of Technology, Department of Physics and Astronomy, Kemigården 1, 41296 Gothenburg, Sweden; § 531195 University of Trento, Department of Physics, Via Sommarive 14, 38123 Trento, Italy; ∥ European Centre for Theoretical Studies in Nuclear Physics and Related Areas (ECT*), Bruno Kessler Foundation (FBK), Strada delle Tabarelle 286, 38122 Trento, Italy; ⊥ Trento Institute for Fundamental Physics and Applications (TIFPA-INFN), Via Sommarive 14, 38123 Trento, Italy

## Abstract

Determining the 3D structure of proteins is a long-standing challenge in the life sciences and is of paramount importance for understanding fundamental biological processes and advancing pharmaceutical development. Experimental techniques such as X-ray diffraction, nuclear magnetic resonance (NMR) spectroscopy, and cryo-electron microscopy (cryo-EM) have been instrumental in determining numerous protein structures. However, these methods are not universally applicable, and new complementary approaches are still needed. Atom probe tomography (APT), a form of mass spectrometry capable of reconstructing the 3D chemical composition of materials with near-atomic resolution, has been proposed as a potential tool for the structural analysis of proteins. Although recent progress has been made, no existing methodology has yet demonstrated both high throughput and reproducibility. In this work, we apply deep-UV laser-assisted APT to study green fluorescent protein (GFP) embedded within an amorphous silica matrix. This approach enables the detection of several thousand proteins within a single measurement, representing a significant advancement toward structural analysis of proteins by APT. To assess whether the embedding procedure preserves the structural integrity of proteins, we combined experimental measurements with molecular dynamics simulations, which strongly suggest that the initial stages of silica encapsulation do not disrupt the native folding of GFP. Overall, these findings demonstrate the potential of deep-UV laser APT as a promising method for structural biology.

## Graphical abstract

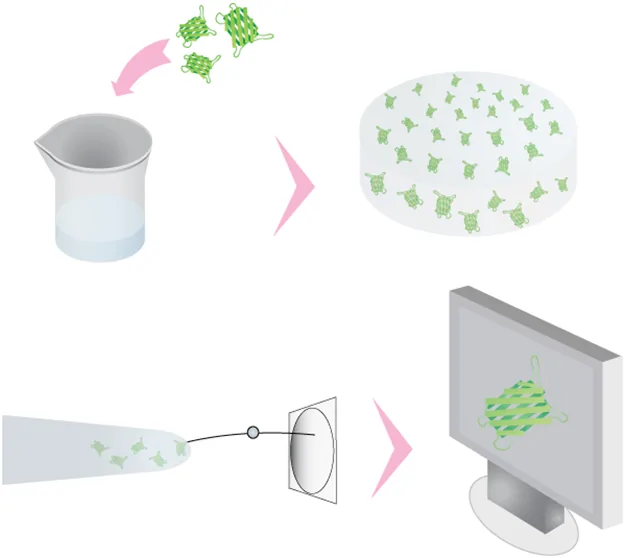

## Introduction

Knowledge of the 3D structure of proteins, commonly referred to as their tertiary structure, is essential for understanding and controlling their function, and is of fundamental importance in biological and medical research. This is also critical for elucidating disease mechanisms and developing treatments, since the interactions between different proteins as well as between proteins and other compounds are governed by their 3D structures.^1^ While the chemical composition and amino acid sequence of proteins generally are well-known and readily determined, this information alone does not predict which tertiary structure the protein will adopt upon folding. Additionally, protein structures are dynamic and sensitive to their environment,^2^ and as such, observations of individual proteins would enable significant progress in understanding biological processes.

Several analytical techniques are available for the determination of protein structures. Historically, X-ray diffraction was the first technique ever employed and is still widely used, although it requires the proteins to be crystallized to obtain structural data.^3^ Nuclear magnetic resonance (NMR) spectroscopy has been used to study the chemical environment of the constituent atoms in biomolecules.^4^ More recently, cryo-electron microscopy (cryo-EM) has emerged as a widely used technique, dramatically increasing the number of resolved protein structures.^5^ However, none of these techniques can provide complete structural information for all classes of proteins, highlighting the need for complementary techniques. Particularly, membrane proteins, which are located in the lipid bilayer of cells, remain elusive to conventional techniques,^6^ despite their crucial role in numerous diseases.^7^ In recent years, computational methods have driven major advances in structural biology, most notably through deep learning models such as AlphaFold.^8,9^ However, these approaches rely on experimental data, both to validate the accuracy of their structural predictions and to expand the underlying training data sets.^10^

Atom Probe Tomography (APT) is a powerful characterization technique used in materials science to characterize metals, semiconductors, nanomaterials, and geological materials.^11^ Fundamentally, APT is a mass spectrometry technique that, in addition to chemical composition, provides the 3D information on the spatial distribution of the elements within a sample at potentially subnanometer resolution, depending on the material studied and analysis conditions.^12^ The underlying principle of APT is based on the controlled field evaporation of surface atoms from a needle shaped specimen (typically less than 100 nm in diameter) exposed to a strong electrostatic field. The evaporated ions are projected onto a 2D position sensitive detector while recording their time-of-flight to determine elemental identity and spatial origin.

Owing to the potential of the technique’s inherent properties, significant efforts have been made to enable the study of biological materials using APT throughout the years,^13^ beginning during the development of its predecessor, field ion microscopy.^14,15^ These efforts continued with studies of organic macromolecules during the early days of APT,^16,17^ followed by work such as investigations of solidified ferritin after the introduction of laser assisted APT and focused ion beam (FIB) based specimen preparation protocols.^18^ However, due to the technical requirements of APT, namely that the samples must withstand ultrahigh vacuum, cryogenic temperatures, and strong electrostatic fields, the technique has mainly been practically applied to hard biological materials such as bones and teeth.^19^ Characterization of soft biological materials and biomolecules has proven more challenging due to the difficulty of preparing specimens that can withstand these conditions while retaining their native state. To address this, several strategies for sample preparation have been explored, including cryogenic specimen preparation from aqueous organic molecular solutions,^20–23^ trapping of target biomolecules in solutions on an APT specimen using graphene,^24^ deposition on presharpened needles,^25^ and embedding them in resins.^26^

In this work, we have further developed and used a sample preparation protocol in which proteins are embedded in a silica matrix to enable APT analysis. This approach has been shown to be able to preserve the native state of proteins while replacing the surrounding water with a solid matrix suitable for APT examination.^27^ Supporting the experimental work, we employed molecular dynamics (MD) simulations to assess whether proteins retain their structural integrity throughout the early stages of the silica matrix formation. These simulations provided molecular insights on the inherent stability of the protein fold induced by the surrounding silica precursors, thereby offering a strategy for assessing the effectiveness of the embedding protocol.

While there are examples where SiO_2_ features in, for example, semiconductor components has been studied by APT,^28–31^ analyzing amorphous silica as a bulk material has been challenging using earlier generations of laser-based APT instruments operating at visible wavelengths, with only few reported analyses.^32^ The difficulties are due to the material’s high porosity, poor absorbance in the used laser wavelength regime, in combination with its poor conductivity. However, the newest generation of commercial instruments is equipped with a deep-UV (257.5 nm) laser, using the fourth harmonic wavelength of a Yb-doped fiber laser (1030 nm) compared to previous generation instruments, where the second or third harmonic wavelength of a Nd:YAG laser (1064 nm) is used.^33^ This has significantly improved the yield of APT analyses of amorphous silica, owing to the higher absorption of the laser and more effective heating.^34^ This has in this case enabled the collection of larger data sets and the characterization of numerous individual proteins within a single analysis.

As a model protein, ^13^C- labeled green fluorescent protein (GFP) was used to allow unambiguous identification of protein-originating carbon in the mass spectrum. GFP is a small protein of 28 kDa, i.e., below the commonly reported minimum size required to achieve high resolution structural data using cryo-EM.

Silica embedded protein samples were prepared from a sodium silicate precursor using a previously developed sol–gel method.^27,35^ The highly alkaline sodium silicate solution was passed through an ion exchange column activated with hydrochloric acid to reduce the pH closer to physiological conditions in order to initiate gelling prior to the addition of the GFP solution. The synthesis protocol is schematically illustrated in Figure 1. In this work, APT specimens prepared from these materials were successfully analyzed, demonstrating that data from a large number of proteins can be collected to provide new insights into the field evaporation behavior of biomolecules.

**Figure 1:**
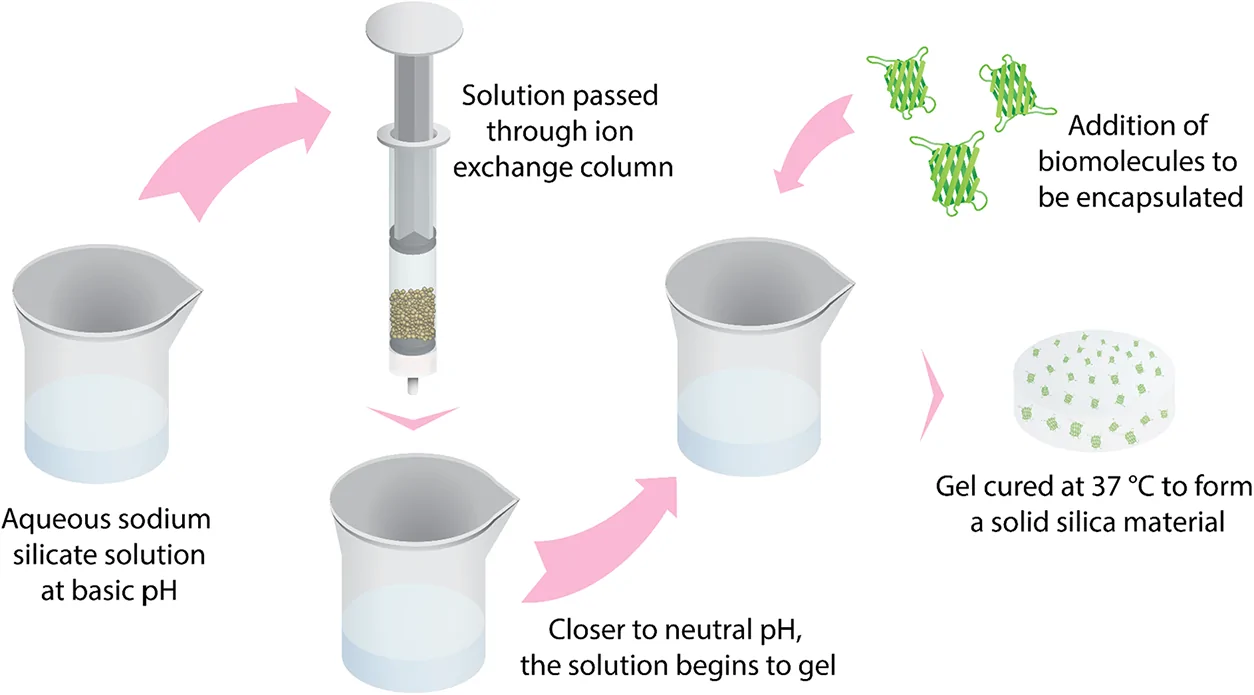
Schematic overview of the protocol employed for synthesis of the silica and subsequent embedding of the biomolecule target.

## Methods

### Silica Embedding of Proteins

The GFP proteins were embedded in a silica matrix from a sodium silicate solution using a previously developed protocol.^27^ A Superclo sodium silicate solution from Sigma-Aldrich containing 7.5–8.5% Na_2_O and 25.5–28.5% SiO_2_ was used, diluted 1:3 with Milli-Q water. ^13^C isotope-labeled Green Fluorescent Protein (GFP) was acquired from AlexoTech AB, Sweden, with a reported purity of >95%.

Silica samples with embedded GFP were prepared according to the sol–gel synthesis procedure shown in Figure 1. An ion-exchange column was setup by using a Superclo Amberchrom 50WX8 ion-exchange resin (Sigma-Aldrich) of which 3 g was placed on top of 1 g of glass wool, separated by a rubber membrane with a small hole cut through it in a 30 mL plastic syringe. NaOH [4M] was passed through the column to regenerate the resin, followed by Milli-Q water, before the resin was activated with HCl [1M]. The column was then adjusted by passing through small amounts of Milli-Q water, until the extrude pH was around 5. Finally, 3 mL of the diluted sodium silicate solution was passed through the column and a pH of approximately 7 was achieved.

100 μL of 13C-GFP solution (1 mg/mL) was immediately added to 200 μL of the sodium silicate solution, as gelling onsets rapidly at neutral pH. Lastly, the solution was cured at 37 °C for at least 24 h to obtain a solid silica material.

### Microscopy and ToF-SIMS Evaluation of Protein Distribution

The obtained silica samples, containing proteins, were embedded in epoxy resin (EpoFix) and ground down to expose a cross section of the material. The material was analyzed using a Zeiss ImagerZ2 in both brightfield and fluorescence microscopy mode to investigate the distribution of GFP in the silica. Fluorescence microscopy was performed using a Zeiss Colibri LED light source operating at 475 nm. A 450–490 nm excitation filter and a 500–550 nm emission filter were used. The same samples were also analyzed by ToF-SIMS in negative mode using an IONTOF TOF.SIMS5 equipped with a bismuth nanoprobe gun to map the distribution of the proteins in the material, operated at a current of 0.4 nA. In addition, a stage bias of 140–180 V was applied and the flood gun was used.

### APT Specimen Preparation

APT specimens were prepared according to the conventional FIB/SEM lift-out protocol, schematically illustrated in Figure 3a, using an FEI Versa 3D with a single Ga^+^ isotope source (69 Da). The regions of interest selected for lift-out were covered with a rectangular protective Pt layer via the gas injection system (GIS) of the instrument using a (methyl-cyclopentadienyl)-trimethyl platinum precursor gas. The trenches surrounding the platinum strip were milled with the stage tilted 30° with respect to the ion beam to obtain a cantilever. The cantilever was then lifted out using an Omniprobe micromanipulator and placed in segments on a silicon flat top post coupon (CAMECA, Madison WI). The lifted-out segments were finally polished into sharp APT specimens by annular milling using the focused ion beam.

### Alternative APT Specimen Preparation

To assess the effect of newly developed strategies that can improve the yield of APT analyses of the silica materials, the finished specimens were coated by FIB in situ redeposition of chromium. The FIB sharpening of the specimens was also performed under cryogenic conditions. The chromium coating was performed in an FEI Versa 3D following the protocol described by Schwarz et al.^51^ Redeposition was performed by milling at 30 pA and 30 kV for 1–2 min at 2 or 4 90° intervals. Cryo-polishing was performed using a Tescan GAIA FIB, on lift-outs mounted on standard LEAP coupons using an FEI Versa 3D.

### APT Analysis

The APT analysis of the silica specimens with embedded proteins was carried out using a LEAP 6000 XR (Cameca, Madison WI) equipped with a deep-UV laser (257.5 nm) under different analysis conditions. The laser pulse energy (LPE) was varied between 1 and 250 pJ, the temperature of the specimen was generally kept at 50 K, the target detection rate was set to 0.5%, and the pulse frequency was controlled via the auto pulse rate control, which was gauged to allow ions with a mass-to-charge state ratio up to 250 Da to reach the detector before the next pulse. The pulse rate history from the analysis in Figure 4 can be found in Figure S7a.

The data sets obtained from the APT measurements were analyzed using APSuite 6.3 (CAMECA, Madison WI). Data reconstruction into 3D atom maps was performed adopting either the tip profile protocol, using SEM micrographs obtained after the FIB/SEM specimen preparation to determine the initial tip radius and shank angle, or by using the voltage profile to determine the evolution of the specimen radius.

### Molecular Dynamics Simulations

The crystal structure of the GFP protein was obtained from the RCSB Protein Data Bank^58^ (entry 2Y0G): The Modeler interface^59^ in UCSF Chimera^60^ was employed to design 24 missing residues—16 at the amino- and 8 at the carboxy-terminus respectively—while the EGFP chromophore was modeled according to the structure reported in Breyfogle et al.^61^ Finally, the coordinates of the silicate trimer (Si_3_O_10_H_7_) were taken from the PubChem database^62^ (CID code: 11966309).

Four simulation scenarios were created with diverse concentrations of the silicate trimer—namely, 0 (*control* scenario), 600, 900, and 1200 mM—in a physiological solution (an example setup is shown in Figure S12): This is consistent with the proposed sample preparation protocol according to the scheme shown in Figure 1, where the silicate trimers are employed as effective proxies for an early stage in the synthesis of the amorphous silica matrix and embedding of the biomolecular substrate. Likewise, the gradient of the silica precursor mimics the steady removal of water molecules through the curing process.

Classical, atomistic molecular dynamics (MD) simulations were performed via the GROMACS 2023 software,^63^ in explicit TIP3P water^64^ and at a 150 mM concentration of sodium chloride. A system equilibration protocol was followed, which included the following: (i) 50,000 energy minimization steps by the steepest descent algorithm; (ii) a 1 ns thermalization in the NVT ensemble at *T* = 298 K (the coupling constant of the Berendsen thermostat^65^ was set to τ_T = 0.1 ps); (iii) a 2 ns volume equilibration in the NPT ensemble at *P* = 1 bar (the coupling constant of the Berendsen barostat and the water compressibility are set to τ_*P* = 2.0 ps and 4.5·10^–5^ bar^–1^ respectively). Throughout the equilibration stage, the heavy atoms of the solute molecules were restrained by a force constant of 1000 kJ/(mol·nm), and an integration time step (*t*_step) of 1 fs was applied. A final unconstrained NPT equilibration of 5 ns was performed, followed by three 500 ns MD replicates totaling 1.5 μs per system. For the latter, we employed a stochastic velocity-rescale thermostat^66^ (τ_*T* = 0.1 ps, *T* = 298 K) and a Parrinello–Rahman barostat^67^ (τ_*P* = 2.0 ps, water compressibility = 4.5·10^–5^ bar^–1^, *P* = 1 bar), associated with a time step *t*_step = 2 fs.

The GFP and the silicate trimer were modeled via the Amber ff14SB^68^ and PolCAFF^69^ force fields respectively, and the Joung-Cheatham corrections^70^ were adopted to treat the monovalent salt. All covalent bonds involving hydrogen atoms were constrained by LINCS,^71^ and long-range dispersion corrections were applied for the energy and pressure calculations.

Iso-density maps of the adsorption of silicates on the surface of GFP were generated using the VMD 1.9.4 ^72^ interface with a grid resolution of 1 Å. Lastly, the GROMACS 2023 analysis toolkits were employed to compute the root-mean-square fluctuation (RMSF), as well as to track the secondary structure propensity of the GFP residues throughout the MD trajectories.

## Results and Discussion

The presence and distribution of proteins in the silica samples were verified through the characteristic yellow-green color and fluorescence of GFP (Figure 2, Figure S1). The fluorescence intensity of GFP is quenched when the protein is denatured,^36^ serving as an indicator that preservation of fluorescence reflects a maintained chromophore and thus indicates a stable protein structure. Additionally, isotope-labeling enabled the use of time-of-flight secondary ion mass spectrometry (ToF-SIMS) to distinguish protein-rich regions in the sample prior to the FIB process. It was generally observed that the proteins were inhomogeneously distributed in the material. At a pH around 8–9, the proteins tended to migrate toward the upper region of the silica sample, creating a GFP rich zone particularly suitable for targeted APT specimen preparation (Figure S2). GFP is known to resist denaturation across a broad range of pH values,^37^ making the slightly basic environment less of a concern. The sample preparation method used is tunable and can be used to embed proteins in silica over a wide range of pH values, depending on which conditions are most suitable for stability and desired distribution of a given protein. At pH values closer to neutral, GFP was distributed more uniformly within the bulk of the silica, though still exhibiting localized regions of higher concentration, as observed by optical (Figure 2a_1_), electron (Figure 2a_2_) and fluorescence microscopy (Figure 2a_3_,a_4_). The higher concentration regions were found to have a different morphology compared to the surrounding matrix (Figure 2b_1_) and correlatively confirmed to be protein containing with ToF-SIMS (Figure 2b_2_). The variation in morphology was also confirmed to not only be a surface feature by FIB-SEM cross sectioning (Figure 2c_1_ and c_2_).

**Figure 2:**
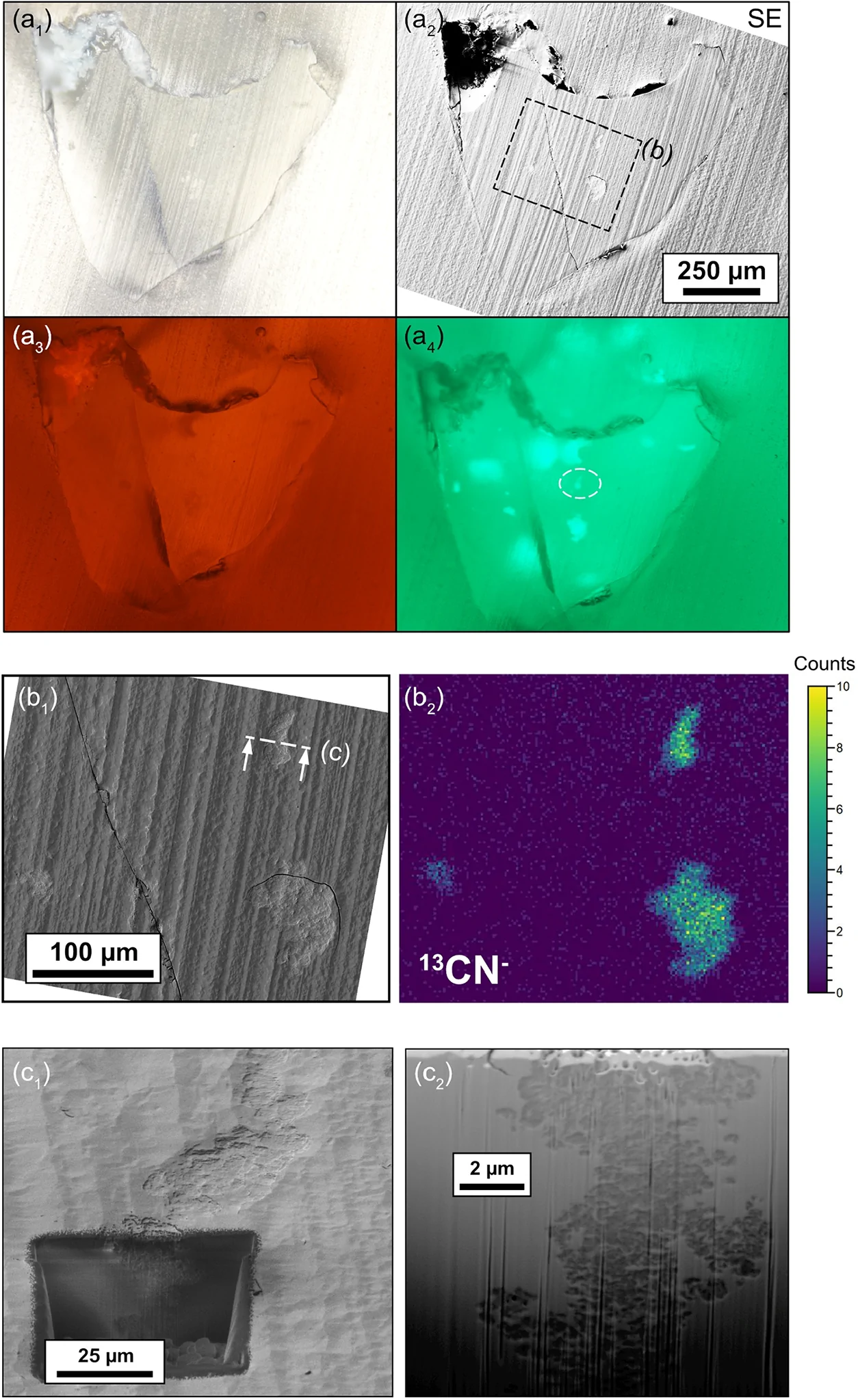
(a_**1**_) Optical bright-field microscopy image of the cross section of a silica sample with GFP embedded in epoxy resin; (a_2_) Scanning Electron Microscope (SEM) micrograph of the same area; (a_3_) fluorescent microscopy image of the same area using a 470 nm excitation wavelength and 525–550 nm emission filter, (a_4_) fluorescent microscopy image of the same area using a 546 nm excitation wavelength and 575–640 nm emission filter. (b_1_) SEM micrograph of the area highlighted in (a_2_), (b_2_) ToF-SIMS mapping of the region highlighted in (a_2_). (c_1_) and (c_**2**_) SEM images of FIB cross sections in the region outlined in (b_1_).

APT specimens were prepared from the protein-rich regions using a conventional FIB/SEM lift-out protocol (Figure 3a).^38^ The selected regions of interest exhibited distinct morphologies compared to the surrounding bulk (Figure 3b,d_1_), and the presence of GFP in the eventual lift-out segments mounted on flat top posts was confirmed by fluorescence microscopy (Figure 3c). The fluorescence was still observable after ion beam milling (Figure 3d_2_–d_3_), confirming that the fluorophore inside the GFP could be preserved during the APT specimen preparation.

**Figure 3:**
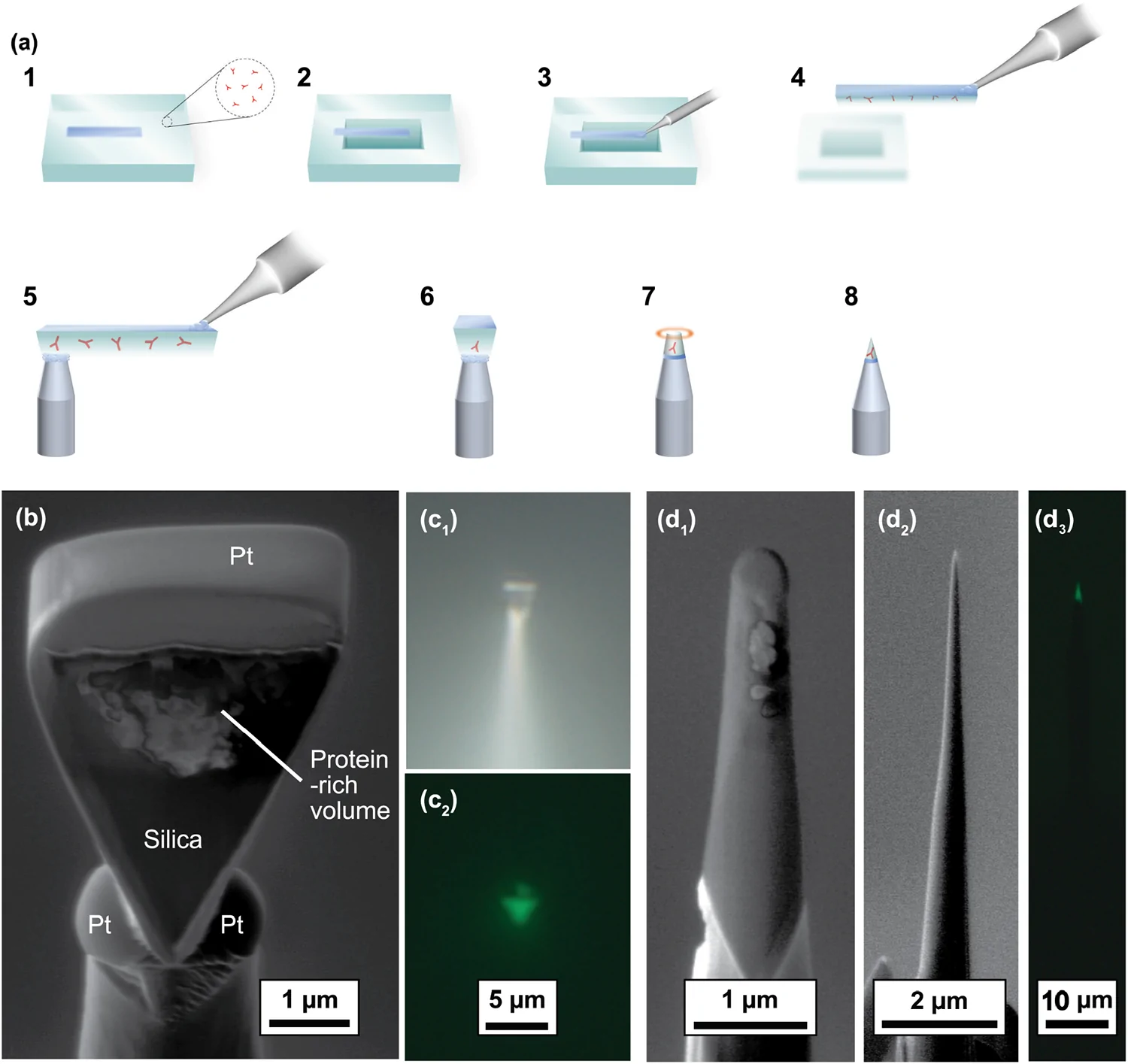
(a) Schematic representation of the APT lift-out process used to obtain specimens from a silica sample containing the embedded proteins. A region of interest (ROI) was identified and coated with a protective Pt-strip using the Gas Injection System (GIS) in the FIB/SEM. The FIB was used to mill trenches and make a cantilever that is lifted out and placed in sections on flat top posts. The lift-out segments were then milled using the ion beam into APT-ready specimens with diameters below 100 nm. (b) Lifted-out section of the material mounted on a presharpened flat top post. The region with high concentration of protein is visible under the platinum capping layer. (c_1_) Brightfield and (c_2_) fluorescence microscopy images of a lifted-out section of the sample as the one shown in (b). (d_1_) SEM micrograph of the sample in (b) during the annular milling; (d_2_) and as a sharpened APT specimen; (d_**3**_) fluorescence microscopy image of an APT specimen after final sharpening.

APT analyses of the silica samples containing GFP labeled with ^13^C yielded complex mass spectra dominated by peaks originating from the silica matrix. The highest count signals corresponded to Si^2+^ at 14 Da and O^+^ at 16 Da, followed by Si^+^ (28 Da), O_2_^+^ (32 Da), SiO^+^ (44 Da) and SiO_2_^+^ (60 Da). Na^+^ is also detected at 23 Da, originating from the sodium silicate precursor used to form the silica matrix. In the APT analyses, carbon was detected at atomic concentrations up to 10% in clusters throughout the analyzed volume. The carbon signal was predominantly observed at 13 Da, at intensities up to ten times higher than the signal at 12 Da (i.e., >90% ^13^C, <10% ^12^C), confirming that the detected organic material originated from the isotope-labeled protein rather than contamination, which would exhibit natural isotope abundances (99% ^12^C, 1% ^13^C). In analyses of nonlabeled proteins embedded and analyzed under similar conditions, no deviation from the natural isotope abundances is observed (Figure S4). It should be noted that in these measurements is uncertain if the organic material is of protein origin. Based on that, the signal at 13 Da is assigned to ^13^C^+^ rather than ^12^CH^+^. In over 10 analyses from different samples, a ^13^C-to-^12^C-ratio over 5 was measured, these analyses were considered successfully and included in this work. Based on the GFP molecular structure (number of carbon atoms) and the instrument detection efficiency, the total number of ^13^C ions detected in the longest run corresponds to up to approximately 10,000 protein molecules. This is attributed to the higher yield of the bulk silica matrix when using a 257.5 nm laser assisted APT system, compared to previous instrument generations with longer wavelengths.^34^ As a comparison, in a previous study of proteins embedded in the same type of matrix analyzed with green-laser-assisted-APT, no more than a single protein was observed in a measurement.^27^ A section of the mass spectrum and the corresponding atom map from one successful measurement are shown in Figure 4 (the complete mass spectrum is available in Figure S5). Owing to the amorphous and porous nature of the silica material, the detection rate during the analysis was nonuniform. As automated voltage control that aims to keep the detection rate constant was used, the resulting voltage profiles were uneven and unsuitable for a voltage-based reconstruction of the entire analysis. The detection rate and voltage history of the analysis reconstructed in Figure 4 can be found in Figure S7. Therefore, accurate spatial reconstruction is not possible with conventional reconstruction protocols. The reconstructions, such as in Figure 4a, were performed using the tip geometry, determined from SEM micrographs acquired during the FIB/SEM specimen preparation (Figure S3).

**Figure 4:**
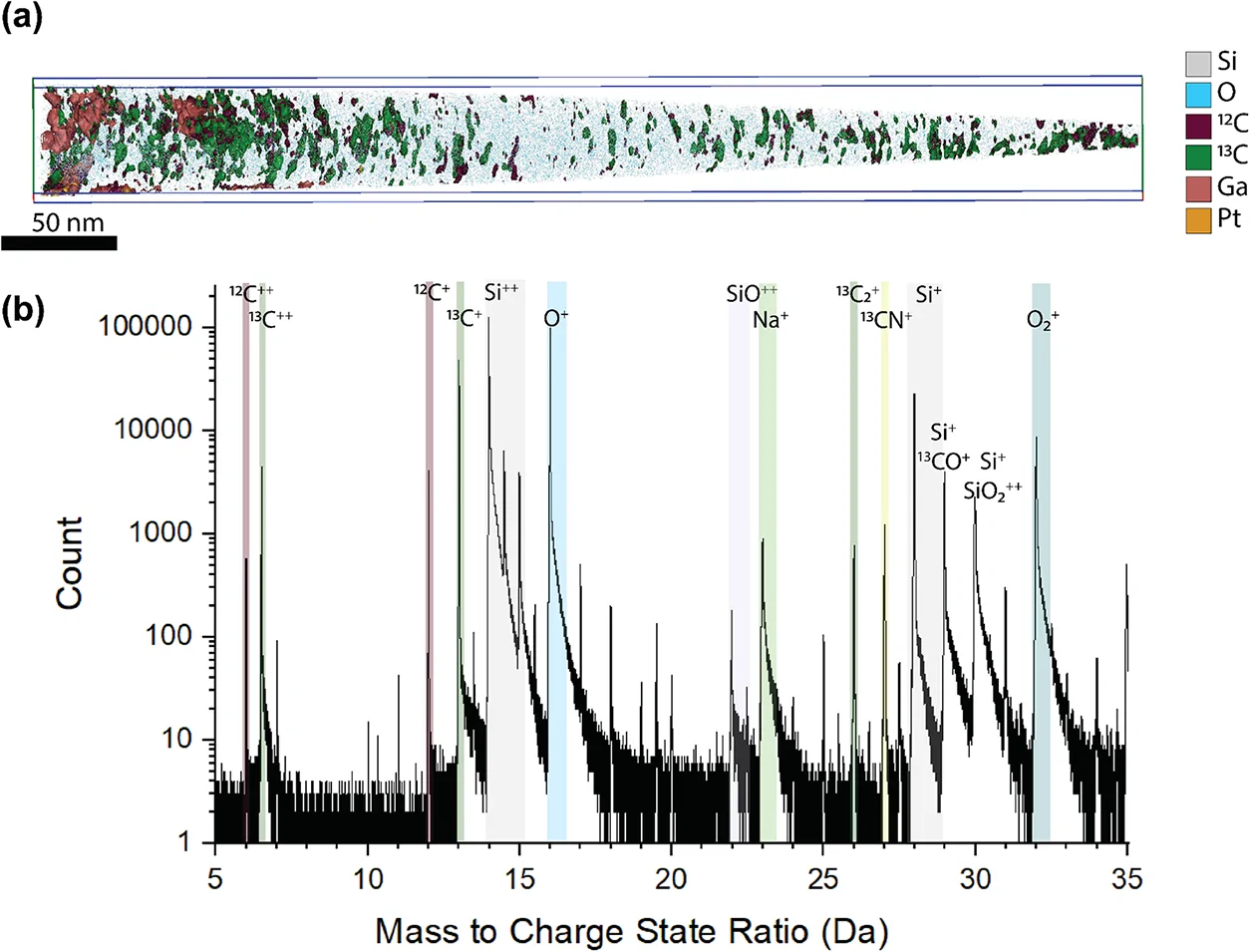
(a) Atom map of a reconstructed APT analysis, carried out with the LPE set to 250 pJ, of a silica sample containing ^13^C-labeled GFP. Silicon atoms are shown as gray dots and oxygen as blue dots. ^12^C, ^13^C, Ga, and Pt are shown as isoconcentration surfaces at 2, 20, 5, and 2 atom % respectively. (b) Segment of the mass spectrum from the analysis in (a), highlighting the identity of the main peaks.

The ^13^C was inhomogeneously distributed, at times forming discernible regions where approximately 500 atoms could be isolated, which closely matches the expected 1134 carbon atoms in a single GFP, given a detection efficiency of 52%. However, larger agglomerations of ^13^C with varying numbers of detected atoms were also observed, demonstrating aggregation of the proteins during embedding in the silica matrix. The observed shape of the proteins strongly depends on the analysis parameters, especially on the laser pulse energy (LPE) and consequently, on the electrostatic field at the specimen surface. At lower electrostatic fields (higher LPEs), ^13^C often appeared as thin layers following the hemispherical shape of the reconstructed specimen, as can be seen in Figure 5a. Likely, this is mainly caused by known APT trajectory aberrations, as void-like features with a lower evaporation field than the surrounding matrix may appear as flattened.^39^ This is explained by preferential evaporation where the majority of the protein material evaporates in short succession over the surrounding matrix. As depth coordinate in the reconstruction is based on the order of detection, the shape will be flattened. The rounded shape following the hemispherical specimen surface is exaggerated by the applied reconstruction algorithm. Reported values for the evaporation field of organic materials such as proteins are lower than for silica.^40,41^ This difference in the evaporation field has an effect at both high and low fields. At lower fields, evaporation of ionic species from proteins may occur preferentially over silica at the evaporation interface, rather than as embedded within the silica matrix, thereby contributing to the observed flattened shape of the protein and negatively affecting the spatial accuracy of the analysis. At higher fields, i.e., lower LPEs, the number of detected ^13^C ions decreased compared to matrix ions, which also reduced reliability of the APT measurement (Figure 5b). The decreased amount of carbon detected indicates losses caused by preferential evaporation of the relatively lower evaporation field carbon as the electrostatic field increases to maintain field evaporation of the silica matrix when the thermal effect is reduced. This reduction can be attributed to losses caused by multiple evaporation events of protein-originating ions, an effect called detector pile-up, where several ions are evaporated in a single pulse, causing losses due to the dead zone of the detector after one detection event.^42,43^ These losses can be inferred from multiple evaporation correlation histograms, while they are less informative for reflectron-equipped instruments, delayed coevaporation was found to be more pronounced for carbon than the silica (Saxey plots, see Figure S6).^44^ The measured multiplicity increases with increasing field (Figure S8), suggesting this effect contributes to the lower amount of ^13^C detected. The increased fraction of multiple events at higher field conditions can be explained by evaporation of multiple ions at a given pulse, but possibly also the dissociation of molecular ion fragments to smaller ones, or atomic ions, which would further deteriorate the spatial resolution.^45^ The lower detected fraction of ^13^C may also be attributed to preferential evaporation^46^ of the lower evaporation field of organic materials. A higher standing voltage is required to maintain the overall evaporation rate at low LPE, which may increase the number of carbon atoms evaporating between pulses and thus hidden in the spectral background; this, however, would cause a higher background count which is not as clearly discernible (Figure S8). The LPE-dependent detected amount of carbon is illustrated in Figure 5c and variations in multiple events and background levels for the data points are provided in Figure S8. Based on the observed behavior, optimal analysis conditions appear to be higher LPEs. Above 100 pJ, carbon losses appear to be sufficiently suppressed, and higher pulse energies can enable longer analyses, which allow for collection of more data. An LPE of 250 pJ seemed to allow for collection of large data sets with low enough carbon losses as to allow for protein-originating clusters to be discernible in the reconstructed data.

**Figure 5:**
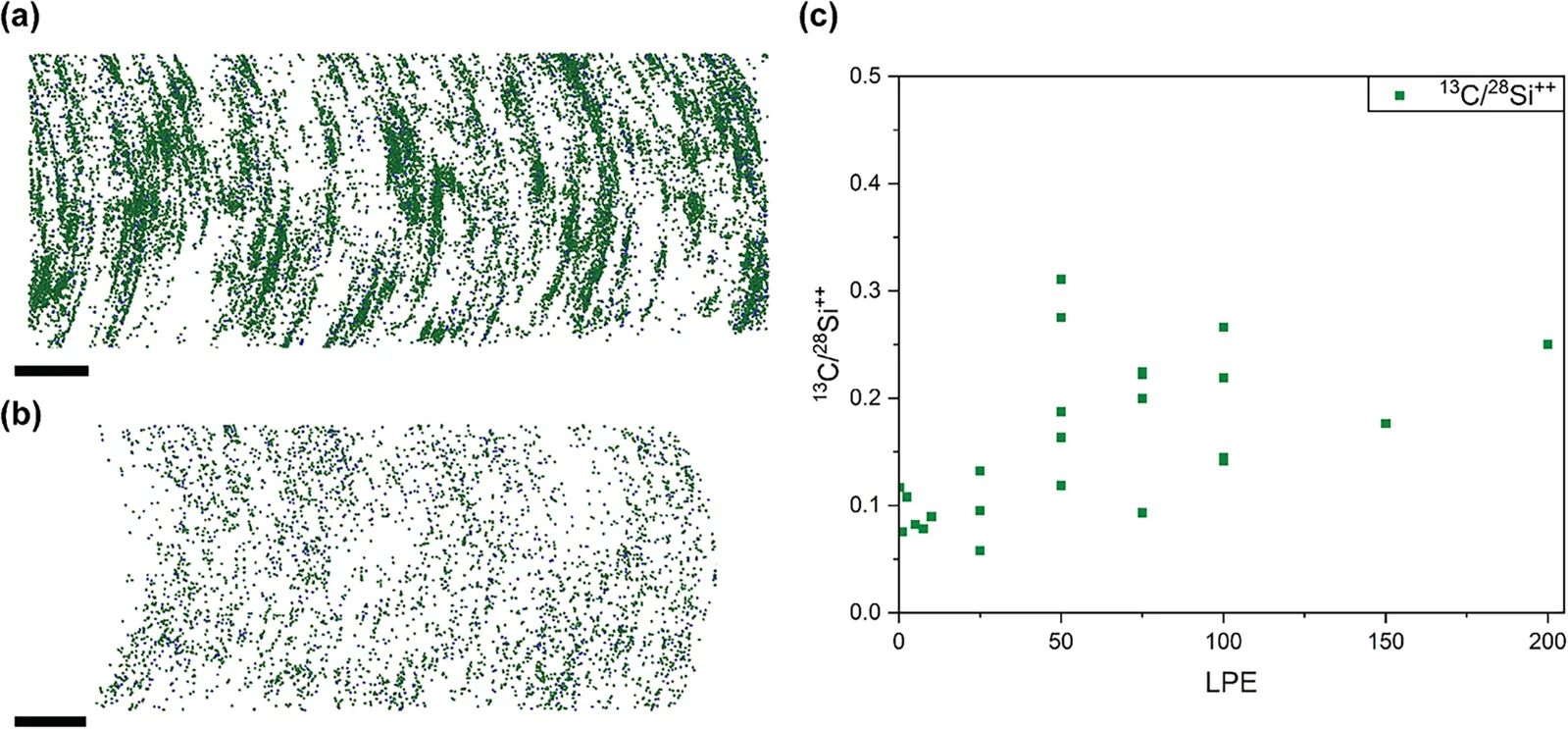
Atom map segments of analyses at (a) 250 pJ and (b) 10 pJ. Segment sizes are 20 × 10 × 50 nm. Scale bar: 5 nm. ^13^C^+^ and ^13^C^2+^ are shown as green and blue spheres, respectively. Similar segments for 50 and 2.5 pJ are shown in Figure S10. (c) The measured ^13^C/^28^SI^+2^ ratios at various LPEs [pJ] from different analyses where the ^13^C/^12^C ratio was above 5.

A few of the clusters shown in Figure 4a contain a number of carbon atoms consistent with a single GFP molecule and can be reconstructed to somewhat resemble the known barrel-shaped structure of the protein with an approximate diameter of 2 nm and length of 4 nm.^47^ An example of such a cluster is shown in Figure 6. However, the observed shapes are distorted by the reconstruction protocol, primarily due to the aforementioned trajectory aberrations and evaporation effects.^48^ At higher fields (lower LPEs), the identification of individual proteins in the reconstructed atom maps is further complicated by the reduced contrast between protein-rich and protein-poor regions due to carbon losses, where areas of high ^13^C concentration become less distinct. For a reliable structural analysis, the field needs to, if possible, be tuned to minimize distortions and trajectory aberrations, field evaporation behavior, and carbon loss.

**Figure 6:**
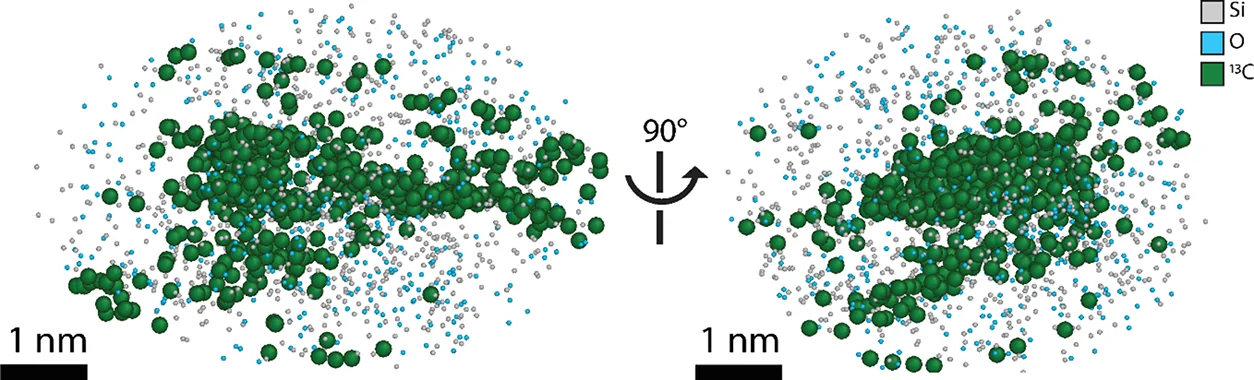
Segment of the atom map in Figure 4a, defined by a “spherical” region of interest (ROI) with the dimensions 4 × 5 × 7 nm, isolating a ^13^C cluster exhibiting size and shape consistent with the known structure of GFP.

To assess whether the observed protein shape was an effect of the silica embedding or APT aberrations, the structural preservation of the GFP native fold upon silica embedding was examined through classical molecular dynamics (MD) simulations. They were performed at varying concentrations of a silicate trimer taken in this work as a proxy of the early stages of the silica matrix formation. Simulations were performed at silica concentrations of 0 M (control scenario), 600 mM, 900 mM and 1.2 M. The results show that GFP is homogeneously embedded within the silica across all silica concentrations, as illustrated by the isodensity maps in Figure 7a–c. Notably, the secondary structure propensity and native fold of GFP^49,50^ were preserved, while fluctuations of residues across the sequence were dampened to a certain extent (see Figure 7d,e), seemingly indicating a lower motility of the protein, and the disordered motifs thereof, induced by silica.

**Figure 7:**
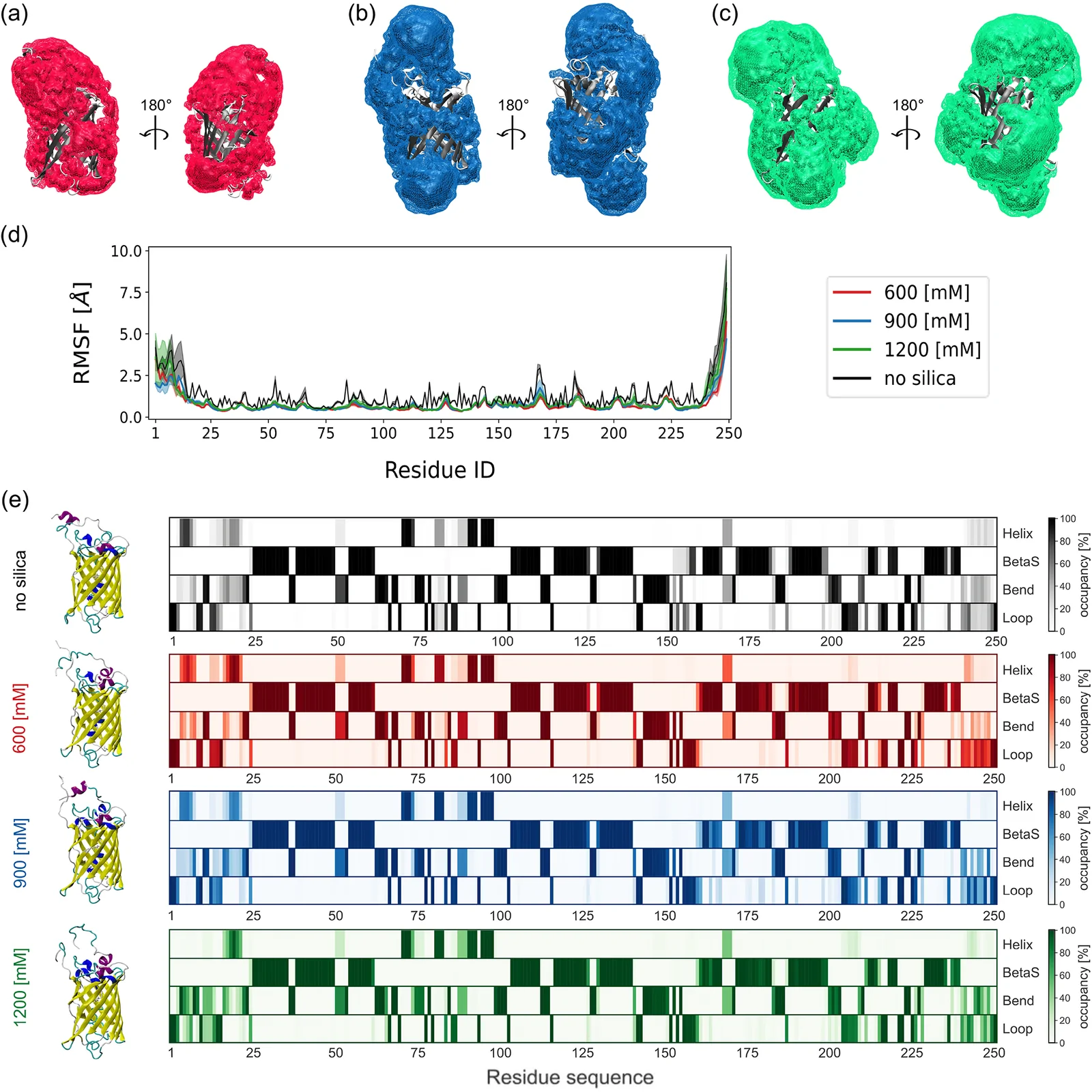
Outcome of the molecular dynamics trajectories of GFP at varying concentrations of a silicate trimer. Iso-density maps showing the homogeneous adsorption of silica on the GFP surface, at silica concentrations of (a) 600 mM, (b) 900 mM, and (c) 1.2 M. (d) Root mean square fluctuations of the α-carbon atoms for each GFP residue relative to the average structure. (e) Secondary structure propensity of each GFP residue in the four scenarios (control, 600 mM, 900 mM, 1.2 M), expressed as the frame-wise percentage along the MD trajectories. The average structures over multiple MD replicates are shown on the left for each system.

In the APT experiments, proteins were primarily detected as atomic carbon in the single charged state, and to a lesser extent in the doubly charged state. Molecular ions such as ^13^C_2_^+^, ^13^C_3_^+^, and ^13^C_4_^+^ were also detected in lower fractions. Although the incorporation of ^13^C into the GFP is >95% according to the manufacturer, ^12^C was also observed in both single and double charge states, and within molecular ions comprising both carbon isotopes. These ions exhibited spatial correlation with atomic ^13^C^+^ according to radial distribution function (RDF) analysis, as shown in Figure 8. Based on the amino acid sequence of GFP, nitrogen and oxygen are expected at atomic ratios of 25 and 30% relative to carbon, respectively. However, the low counts of N^2+^ showed little spatial correlation with carbon. Moreover, the overlap with ^14^Si^2+^ and N^+^ makes detection challenging, but no deviation from the expected natural abundance of the Si^2+^ isotopes was observed. In the analysis with the highest relative amount of ^13^C^+^ detected, a shoulder to the right of the ^14^Si^2+^ peak was observed, matching the mass to charge state ratio of ^14^N^+^ (Figure S11). An excerpt of the mass spectrum is available in the SI. Due to the overlap with the thermal tail of ^14^Si^2+^, quantification of N is unreliable. The ion detected at 27 Da was identified as ^13^CN^+^, which displayed some correlation with ^13^C (Figure 8), suggesting that it originates from proteins. In several measurements, the signal at 29 Da was higher than expected based on the corresponding ^28^Si^+^ signal at 28 Da and the natural isotopic distribution of silicon. This excess can be attributed to overlapping contributions from ^13^CO^+^ and ^29^Si^+^, rendering reliable quantification difficult. Overall, oxygen showed poor correlation with carbon based on the RDF analysis and is therefore attributed primarily to the silica matrix. Furthermore, the RDF analysis should be taken with some caution as the spatial accuracy of the reconstruction is limited, as the reported distances are incorrect and the reconstruction makes the errors more anisotropic. Still, it provides a qualitative indication of which ions are correlated.

**Figure 8:**
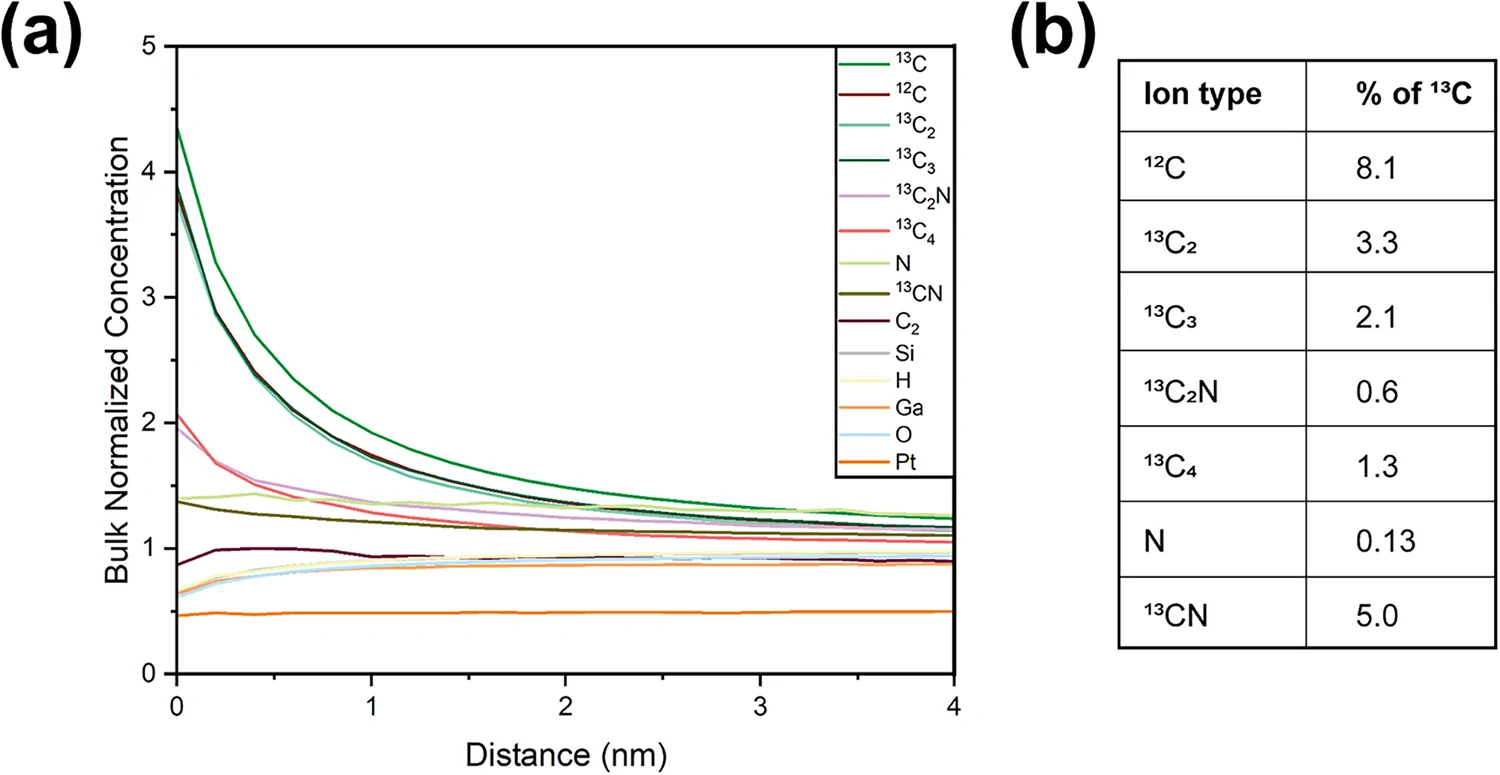
(a) Radial Distribution Function (RDF) of ^13^C ions showing the bulk normalized concentrations of all detected ionic species with atomic ratios above 0.5% with respect to ^13^C. (b) Table showing the atomic concentrations of the relevant ionic species from (a), expressed as a percentage of the ^13^C concentration.

The number and spatial distribution of proteins in the silica matrix varied within and between specimens. Nevertheless, ^13^C-labeled proteins were detected in all specimens prepared from lift-outs taken from identified high protein concentration regions. The accumulation of the proteins in such regions was clearly observed and attributed to the mobility and water solubility of GFP. The segregation of proteins also appeared to be pH-dependent, and under higher pH conditions (8–9), causing them to migrate toward the upper surface of the material during gelling and drying within the confined volume of a well-plate. Despite this inhomogeneity, each analysis contained a sufficient number of individual proteins to potentially enable future use of averaging methods for structural characterization.

In all APT analyses of amorphous silica, the detection rate was uneven throughout the measurement, reflecting the porous nature of the material (see Figure S7b). In an attempt to mitigate this limitation, a recently developed technique, in which sharpened APT specimens are coated *in situ* with a conductive layer by FIB redeposition, was applied,^51^ using Cr. This strategy was expected to reduce microfractures and spikes in the detection rate and to suppress potential carbon surface diffusion by improving thermal conductivity. However, no significant improvement in these effects, nor other detectable differences, were observed compared to uncoated specimens (Figure 9). We observed that the DC voltage required to maintain the set evaporation rate (0.005 ions/pulse) decreased upon transitioning from the Cr capping layer of the specimen into the silica, suggesting that the evaporation field of silica is lower than that of chromium under the experimental conditions employed,^52^ or a contribution from nonthermal field evaporation effects of the interaction between oxides and the deep-UV laser which is not yet fully understood.^53,54^

**Figure 9:**
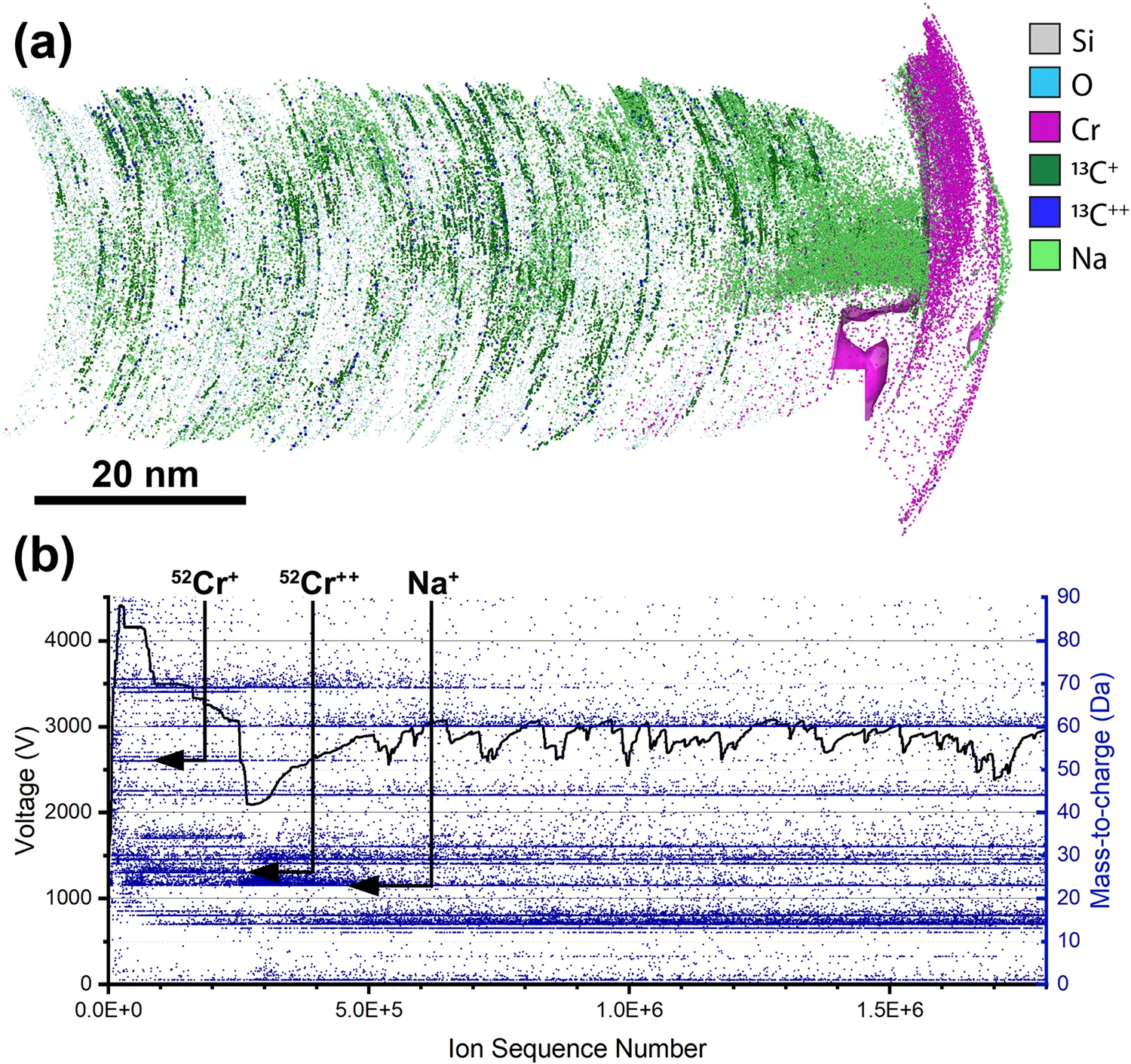
(a) 5 nm thick slice from a voltage-based reconstruction of a cryo-sharpened specimen coated with Cr (magenta), measured at 100 pJ (50 K, 0.5% detection rate). (b) Voltage and mass-to-charge ratio history from the same measurement, displaying the decrease in the applied field after passing through the chromium cap in order to maintain a constant evaporation rate.

It has previously been shown that performing FIB sharpening of APT specimens from a lift-out at cryogenic temperatures can reduce heat-induced damage from the ion beam.^55,56^ This procedure was used here to assess whether ion beam damage to the proteins could affect the analysis. APT measurements of specimens sharpened under cryogenic conditions revealed that sodium had an increased tendency to evaporate at the beginning of the measurement (Figure 10). This can be due to migration to the surface of the tip caused by the electrostatic field, which can be observed for mobile elements such as sodium and lithium.^57^ This observation suggests that some sodium migration occurs inside the specimen induced by the ion beam at noncryogenic conditions, which prevents leaching of sodium when the strong electrostatic field is applied during initialization of APT measurements. During ion milling at cryogenic conditions, no sodium migration occurs, and consequently, sodium is distributed in the silica matrix from which is leached when the voltage is applied. However, no discernible deviation in the evaporation behavior of protein-originating carbon was observed for cryo-sharpened specimens.

**Figure 10:**
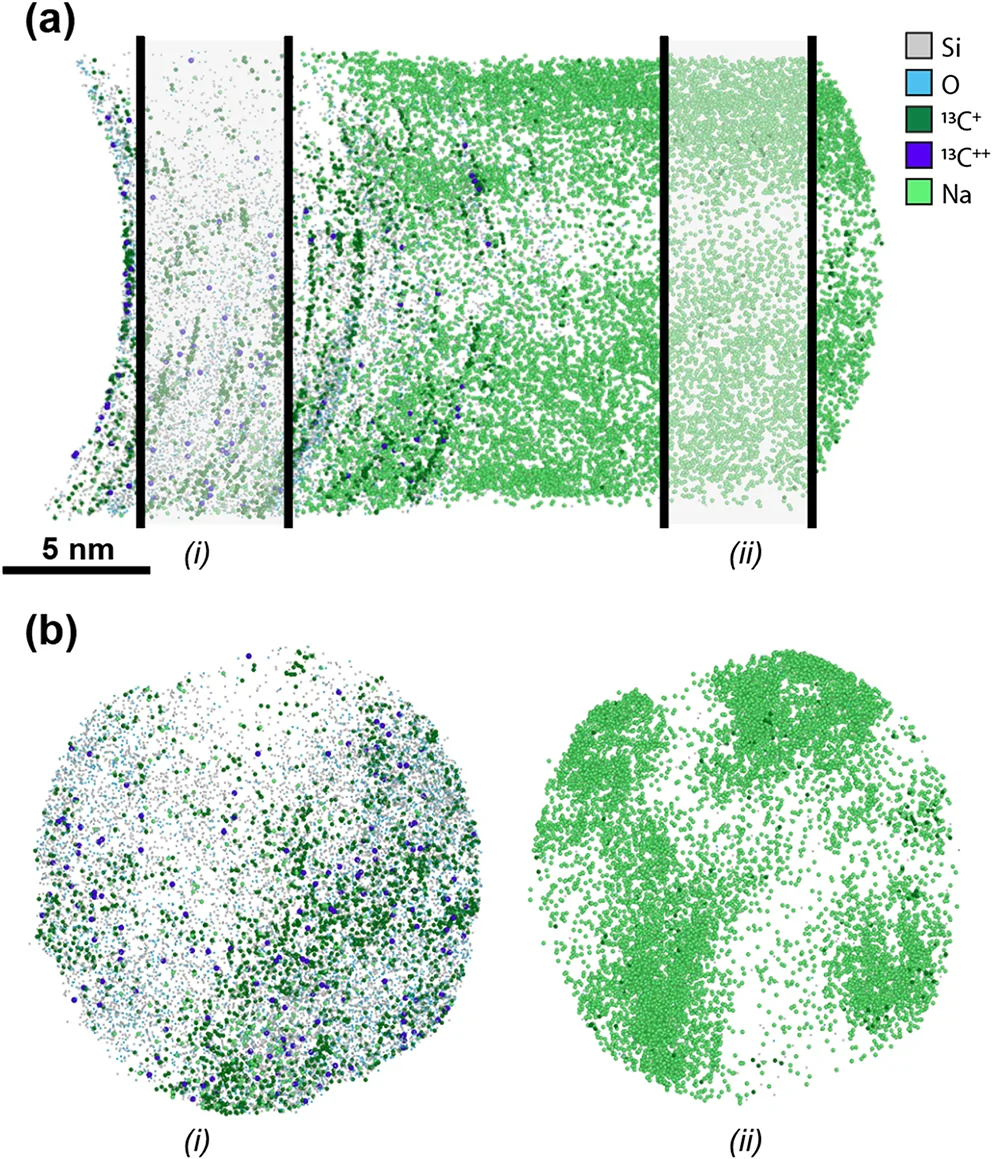
(a) 5 nm thick slice from a reconstruction of a specimen sharpened under cryo conditions, measured at 150 pJ. (b) (i) and (ii) are 5 nm slices viewed along the *z*-direction, from regions displayed in (a) highlighting the preferential evaporation of Na^+^ prior to the evaporation of the protein containing silica matrix.

Since proteins are here primarily detected as atomic carbon and in higher numbers, the potential resolution of the technique is improved compared with previous studies in which molecular fragments dominated the signal.^22–25,27^ These previous studies are predominantly carried out using matrixes requiring a lower electrostatic field for field evaporation. For 3D structural reconstruction, atomic ion signals should enable a theoretically higher spatial resolution; while for applications where sequence information or location of specific groups are of interest, matrix and analysis parameter choice might be tuned to obtain molecular ion signals that allow distinguish and discern of the studied protein’s constituent amino acids. Another potential strategy for such questions might be targeted isotope-labeling of specific groups of interest in the target protein. As carbon is the main element of amino acids, the loss of nitrogen and oxygen should not pose a significant limitation for structural analysis. The use of ^13^C-isotope-labeled proteins allows for a clear distinction of potential contaminants within the analyzed material and is well established in several analytical procedures in the life sciences. Moreover, selective isotope labeling could be used to highlight specific regions of interest within proteins, for example to study active sites relevant for understanding biological processes, diseases, and pharmaceutical development. The silica embedding protocol used here needs to be further developed to avoid agglomeration of proteins and APT analysis conditions optimized to reduce aberrations. Furthermore, proteins with more characteristic 3D structures should be studied to refine and develop new reconstruction protocols specifically for such biomolecules, so that the powerful capabilities of APT can be used to address fundamental questions in structural biology.

## Conclusions

In this work, we have demonstrated that deep-UV laser-assisted APT can be successfully used to capture proteins embedded within an amorphous silica matrix, enabling a far more detailed investigation than previously possible. ^13^C-isotope labeling was employed to unambiguously distinguish between the signal originating from GFP from potential sources of contamination. During sample synthesis, the protein segregated into regions of high concentration and APT analyses of specimens extracted from these regions yielded strong ^13^C signals, a proportion of which formed clusters consistent with the expected number of carbon atoms in a single protein.

A wide range of parameters was evaluated and it was found that higher LPEs give the lowest carbon loss and enable collection of more data. In the reconstructed 3D atom maps, isolated ^13^C-ion clusters whose shape and size somewhat resemble the known structure of GFP were observed, albeit in low numbers compared to the detected amount of ^13^C. Classical molecular dynamics simulations strongly suggested that silica embedding preserves the secondary and tertiary structure of GFP. While further work is needed to refine the current protocol and achieve higher-resolution information, these results demonstrate that APT analyses of proteins can provide data from a large number of individual biomolecules embedded in a matrix, predominantly detected as atomic carbon ions. These observations can further the potential of APT as a method for molecular-scale investigations in structural biology.

## Supplementary Material



## Data Availability

The data used in this study are available from the corresponding author upon request.

## Notes

Additional experimental details, materials and methods, overview SEM and ToF-SIMS data, and APT reconstruction parameters and results; APT results include mass spectrum analysis, multiple event analysis, and additional analysis information such as voltage, detection rate, and pulse rate history for the main analysis in Figure 4; additional APT data associated with the data points in Figure 5; and conceptual molecular dynamics workflow is also presented (PDF)

## #

Max Planck Institute for Sustainable Materials (formerly known as Max-Planck-Institut für Eisenforschung GmbH), Circular Metallurgy and Alloy Design, Max-Planck-Str. 1, 40237 Düsseldorf, Germany
