# Supplementary material for: Silica-Embedded Proteins Enable Higher Yield and Atomic Ion Detection in Deep-UV Laser-Assisted Atom Probe Tomography
Source: Anal Chem. 2026 Aug 14;98(33):24290–302. doi: 10.1021/acs.analchem.6c02770 (PMC13523547; doi:10.1021/acs.analchem.6c02770)
Supplement: Supplementary file 1 [file ac6c02770_si_001.pdf]

Supplementary information

Silica-Embedded Proteins Enable Higher Yield and Atomic Ion Detection in Deep-UV Laser-Assisted Atom Probe Tomography

Authors: Gustav Eriksson[1], David Mayweg[2], Giovanni Novi Inverardi[3,4,5], Francesco Carnovale[3,4,5], Mats Hulander[1], Mattias Thuvander[2], Lorenzo Petrolli[3,5], Gianluca Lattanzi[3,5], Simone Taioli[4,5], Martin Andersson[1*]

[1] Chalmers University of Technology, Department of Chemistry and Chemical Engineering, Kemigården 4, 41296 Gothenburg, Sweden

[2] Chalmers University of Technology, Department of Physics, Kemigården 1, 41296 Gothenburg, Sweden

[3] University of Trento, Department of Physics, Via Sommarive 14, 38123 Trento, Italy

[4] European Centre for Theoretical Studies in Nuclear Physics and Related Areas (ECT*), Bruno Kessler Foundation (FBK), Strada delle Tabarelle 286, 38122 Trento, Italy

[5] Trento Institute for Fundamental Physics and Applications (TIFPA-INFN), Via Sommarive 14, 38123 Trento, Italy

* Corresponding author: martin.andersson@chalmers.se

Table of Contents

Material and Methods 3

Distribution of GFP in silica material 3

APT Data Reconstruction Parameters 4

APT mass spectrum signal identification 4

Multiple event analysis 5

Additional APT results 6

Molecular dynamics 7

References 7

Figures 8

Tables 20

Material and Methods

The materials used for silica sample preparation were a Superclo sodium silicate solution from Sigma-Aldrich containing 7.5 - 8.5 % Na_2O and 25.5 – 28.5 % SiO_2. The solution was diluted 1:3 with Milli-Q water prior to use.

^{13}C isotope labelled Green Fluorescent Protein (GFP) was acquired from AlexoTech AB, Sweden, with a reported purity of >95 %.

The ^{13}C-GFP containing silica samples were prepared using the sol-gel synthesis process described in (1). The ion-exchange column was prepared using a Superclo Amberchrom 50WX8 ion-exchange resin (Sigma-Aldrich) of which 3 g was placed on top of 1 g glass wool separated by a rubber membrane with a small hole cut through it in a 30 ml plastic syringe.

Distribution of GFP in silica material

^{13}C-GFP containing silica samples were embedded using epoxy resin and ground down to expose a cross section of the sample. The material was studied using fluorescence microscopy with a Zeiss ImagerZ2 to investigate the distribution of GFP within the silica. Microscopy images are shown in Figure S1.

Epoxy-embedded silica samples were also analyzed by Time of Flight Secondary Ion Mass Spectrometry (ToF-SIMS) using an IONTOF TOF.SIMS5 equipped with a Bismuth nanoprobe gun. to study the distribution of proteins within the material. Protein-originating ^{13}CN, was found present in silica fragments at the top of the sample, as it had formed during gelling and curing in the confined volume of a well-plate well. ToF-SIMS results together with the corresponding SEM micrographs are shown in Figure S2.

APT Data Reconstruction Parameters

Datasets obtained from APT measurements were analyzed using APSuite 6.2 (CAMECA, Madison WI). Data reconstruction into 3D atom maps was performed using the tip profile protocol, where SEM micrographs obtained during FIB/SEM specimen preparation are used to determine the initial tip radius and shank angle. The SEM micrograph of the specimen analyzed in Figure 4 is displayed in Figure S3.

Relevant reconstruction parameters used were image compression factor (ICF): 1.65, evaporation field: 33 V/nm, with the k-factor generally set to 3.3 but varied between reconstructions in an attempt to resemble the protein shape provided by established characterization techniques.

APT mass spectrum signal identification

In Figure S4, a segment of the mass spectrum obtained from an APT analysis of non-isotopically labelled GFP embedded in silica is shown. The segment is around 12 and 13 Da, with the expected natural abundancies of the carbon isotopes marked. While the origin of this carbon cannot unambiguously be confirmed to be GFP, this indicates that the signal at 13 Da is dominated by $^{13}C^{+}$ without any meaningful contribution from $^{12}CH^{+}$.

The full mass spectrum obtained from the APT analysis in Figure 4 is shown in Figure S5.

Multiple event analysis

The occurrence of multiple events during APT analysis, where more than one detector event occurs between two sequent laser pulses was investigated. In Figure S6 (a), the normalized mass spectra of single and multiple events are compared to elucidate which ion species are predominant in each category. In (b) and (c) of the same figure, correlated evaporation histograms, known as Saxey plots (2), are presented. They illustrate which ion species are evaporating together during multiple events, providing information about the nature of these events.

It was found that ionic species originating from the silica matrix are generally found in relatively larger numbers among single events, along with gallium. On the other hand, platinum and the related organic precursor originating ions are overrepresented among multiple events. The main protein originating peak at 13 Da is not overrepresented among the multiples nor among the singles, however, the peak at 6.5 Da is somewhat overrepresented among the multiples.

As a reflectron equipped APT instrument was used, no molecular dissociation tracks were observed in the correlated evaporation histograms. Some delayed evaporation was observed, associated with the major signals and correlated delayed evaporation of the matrix ions.

Additional APT results

Figure S7, displays additional analysis information from the APT measurement that is reconstructed in Figure 4, namely voltage, detection rate, pulse rate and CSR (field estimate) history. Figure S7c shows that while the $O^+/(O_2^++O^+)$-ratio is relatively constant throughout the measurement, the $^{28}Si^{++}/(^{28}Si^{++}+^{28}Si^+)$-ratio is inversely proportional to the detected ^{13}C-atomic concentration. This indicates that $^{13}CO^+$ overlaps with $^{28}Si^+$, contributing to the 28 Da-signal at higher ^{13}C-concentrations.

In Figure S8, Background and fraction of multiple events for the corresponding data points in Figure 5c is displayed. The data used to construct the graphs in Figure 5c and S8 is presented in Table S1, together with the detected ^{13}C counts corresponding to each data point in the graphs. A general overview of the ^{13}C-CSR-ratio for the analyses, shows an increasing field with decreasing LPE, as expected. In all presented analyses, the $^{13}C/^{12}C$-ratio is above 5, and appear to be dependent on factors such as lift-out location and specimen preparation rather than any analysis conditions. The number of ^{13}C detected in the different analyses is due to changed analysis conditions (LPE) or specimen fracture, which tended to occur prematurely when field conditions were higher.

In Figure S9, the normalized mass spectrum from the regions inside the 30 at. % 13C isoconcentration surfaces in Figure 4 is compared to the normalized mass spectrum of the full analysis. The peaks that are found in a relatively higher number within the regions defined by the isoconcentration surfaces are labeled in the figure.

The highlighted peaks correspond to ^{12}C and ^{13}C in the first and second charge states, as well as $^{13}C_n$ up to n=5. The peak at 19.5 Da is identified as $^{13}C_3^{++}$.

In Figure S10 (a) and (b), segments of APT analyses conducted at the LPE set to 50 and 2.5 pJ LPE respectively are presented similar to Figure 5, showing the carbon evaporation behavior.

The atom maps seemingly follow the same trend as observed in Figure 5, where higher LPEs yield a higher amount of carbon detected, but also exaggerate the phenomenon where carbon seems to diffuse on the surface of the tip. This effect is less prominent at lower LPEs but still occurs, especially towards the edges of the specimen, partly as a consequence of the applied reconstruction protocol. However, less carbon is detected in the latter case, suggesting preferential evaporation between the pulses due to the higher electrostatic field required to maintain the desired evaporation rate at lower LPEs.

In Figure S11, an excerpt around 14 Da from the mass spectrum obtained from the analysis with the highest detected amount of ^{13}C is shown. Here, a shoulder to the right of the main $^{14}Si^{++}$ peak can be seen, matching the mass to charge state ratio of $^{14}N^{+}$.

Molecular dynamics

In Figure S12, the conceptual workflow for the molecular dynamics scenarios is shown.

References

1. Sundell G, Hulander M, Pihl A, Andersson M. Atom Probe Tomography for 3D Structural and Chemical Analysis of Individual Proteins. Small. 2019;15(24):1–6.

2. Saxey DW. Correlated ion analysis and the interpretation of atom probe mass spectra. Ultramicroscopy [Internet]. 2011;111(6):473–9. Available from: http://dx.doi.org/10.1016/j.ultramic.2010.11.021

Figures

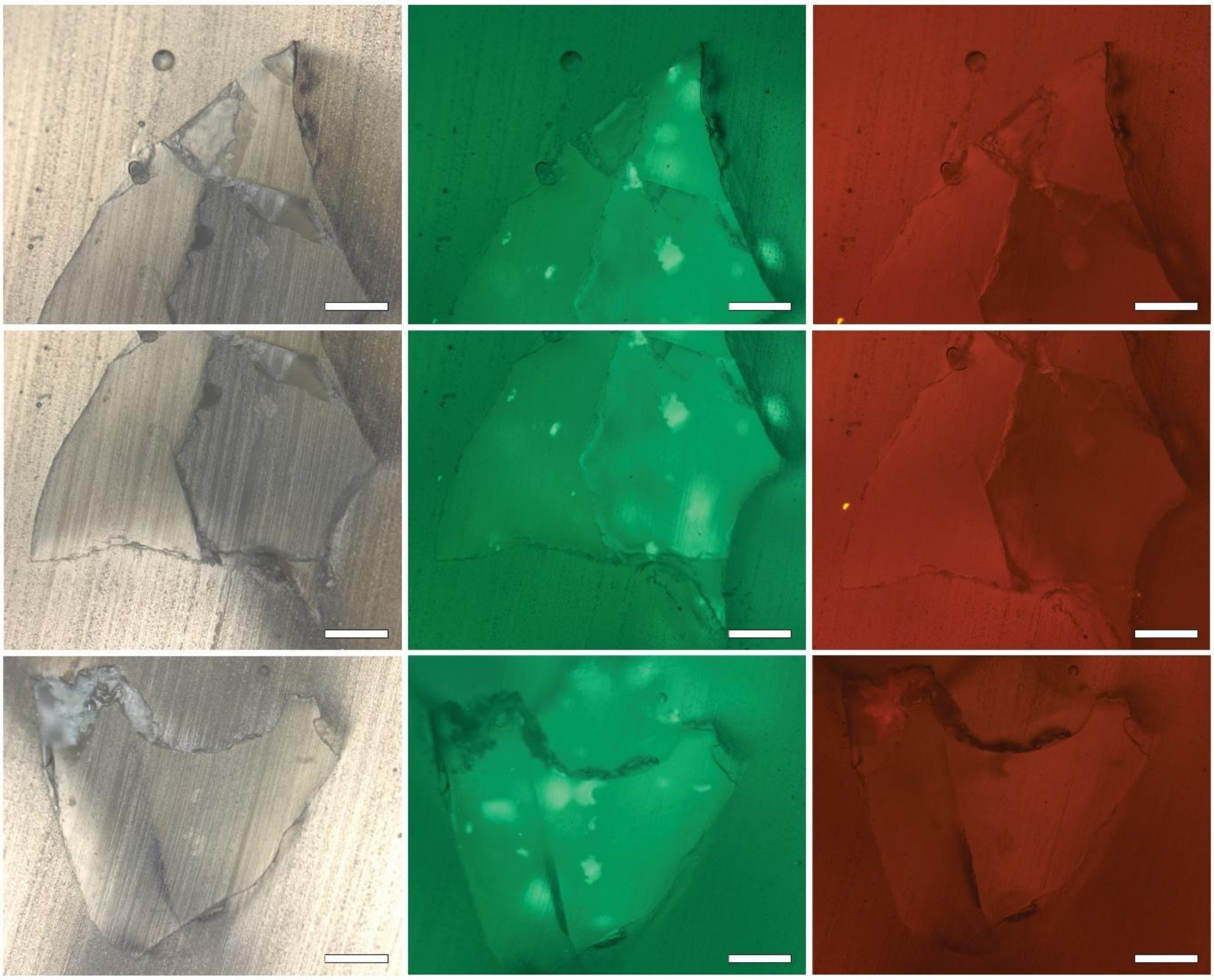

Figure S1: Three samples of silica containing ^{13}C-labelled GFP, embedded in epoxy, imaged by microscopy at 10x magnification. Fluorescence microscopy images of the same regions shown in the middle and to the right. Scale bars: 200 µm.

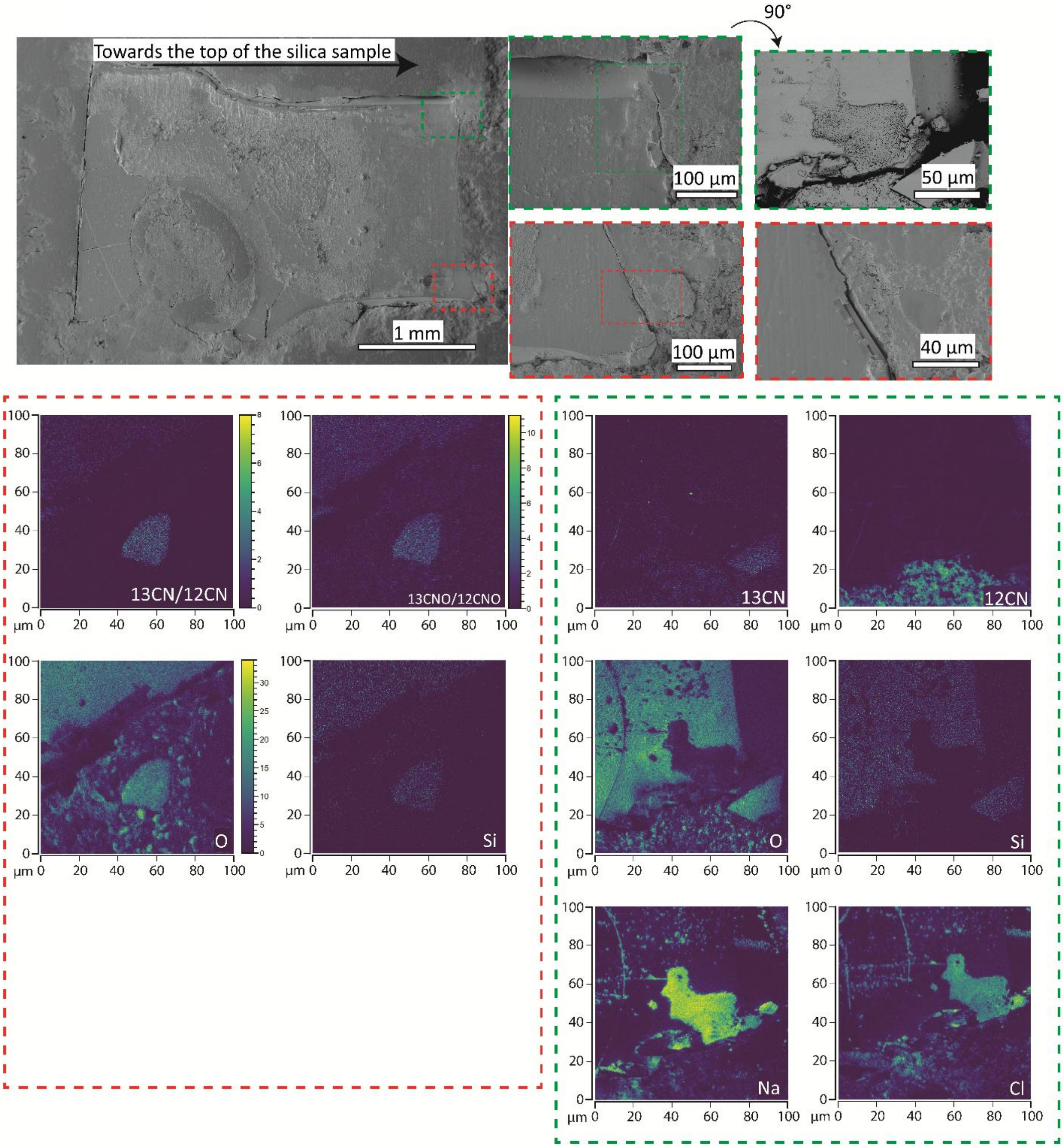

Figure S2: SEM micrographs of an epoxy-embedded silica sample containing ^{13}C-labelled GFP. Two regions at the top of the sample are indicated in green and red. These regions were studied using ToF-SIMS, revealing higher amounts of ^{13}C-containing ionic species in silica fragments at the top of sample, as it had formed during gelling and drying in a well-plate well.

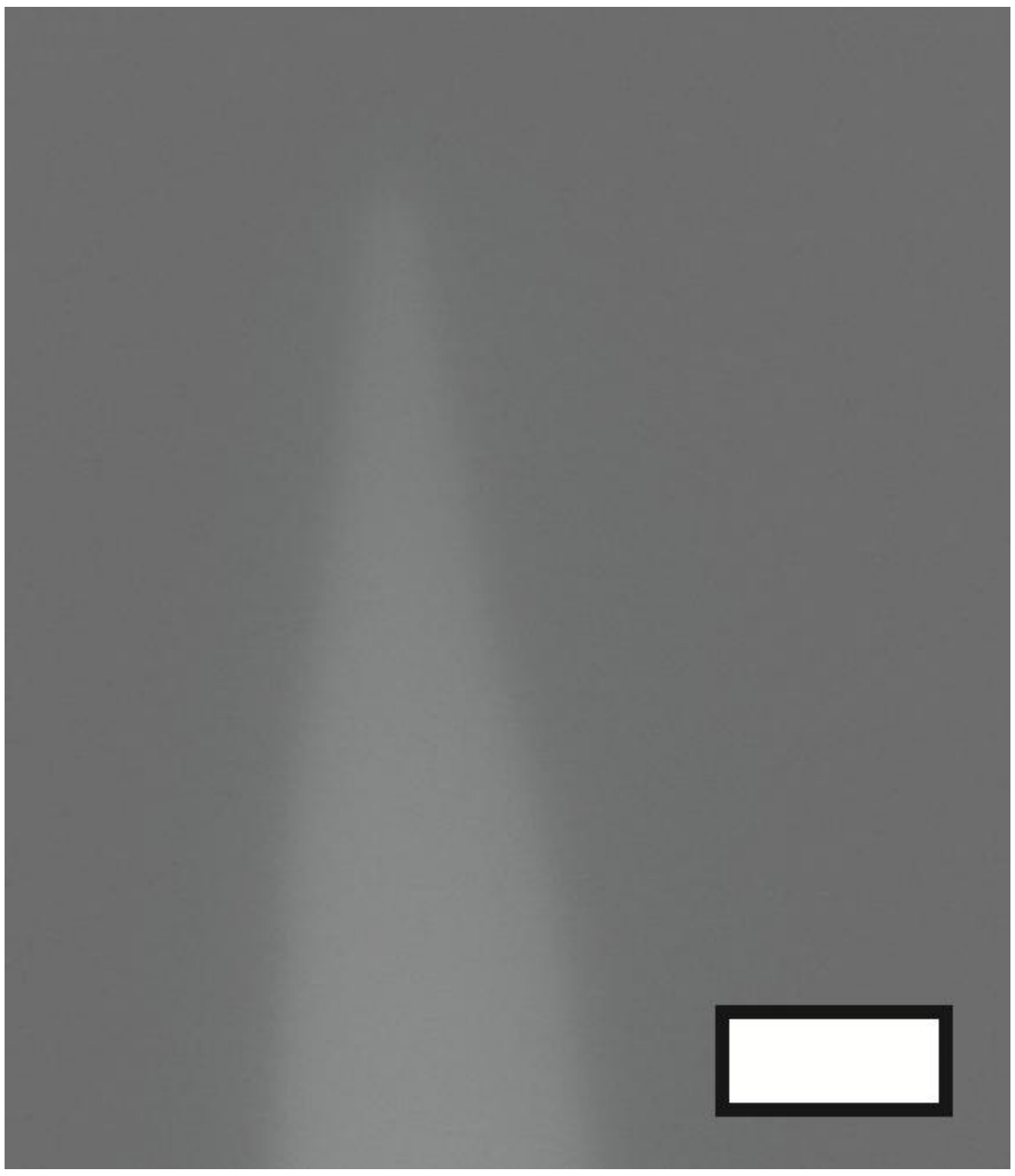

Figure S3: SEM micrograph of the APT specimen analyzed in Figure 4, after specimen preparation prior to analysis. Scale bar: 100 nm.

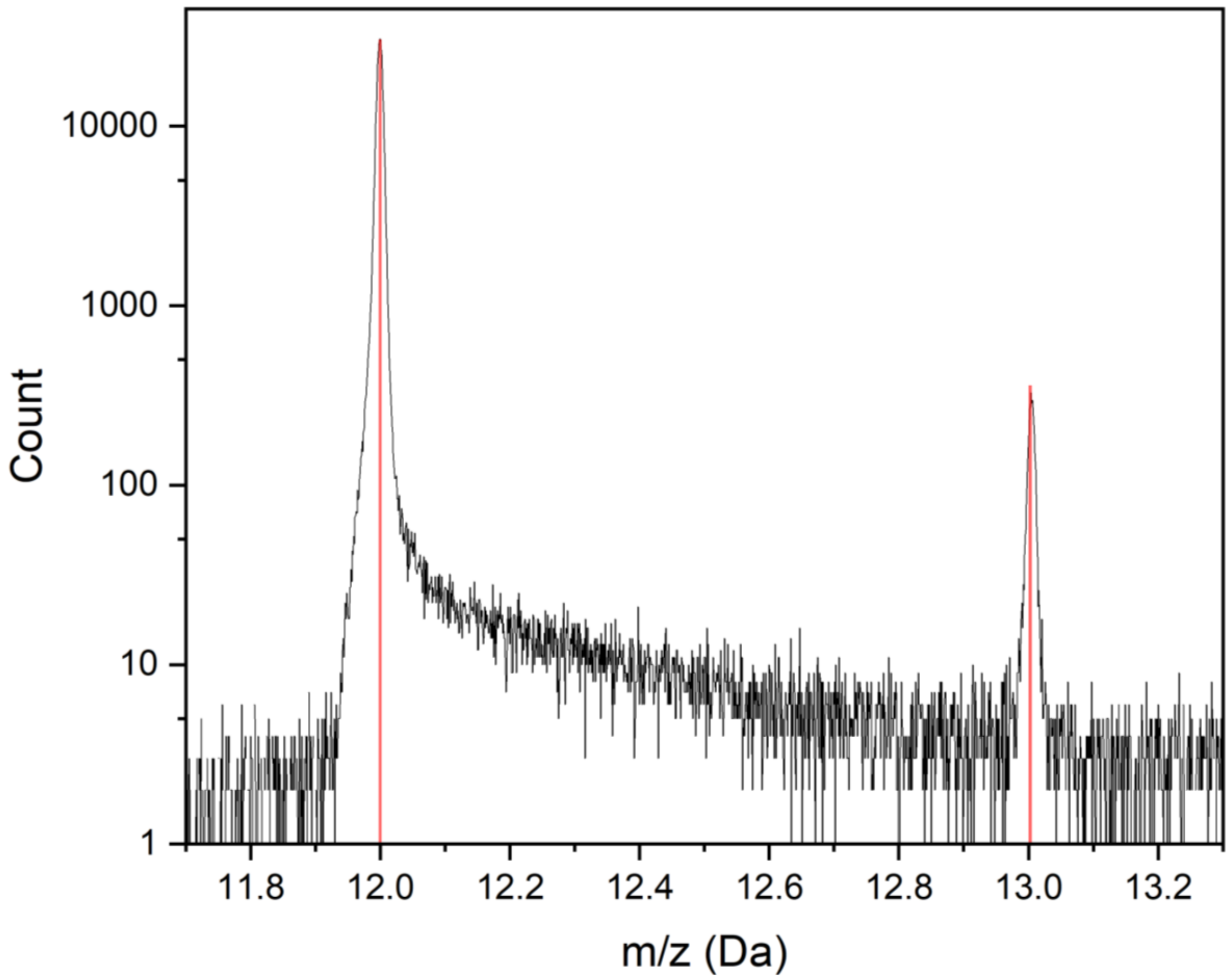

Figure S4: Segment of mass spectrum obtained from APT an analysis of non-isotopically labelled GFP embedded in silica. The relative natural abundancies of ^{12}C *and* ^{13}C *are shown as red bars.*

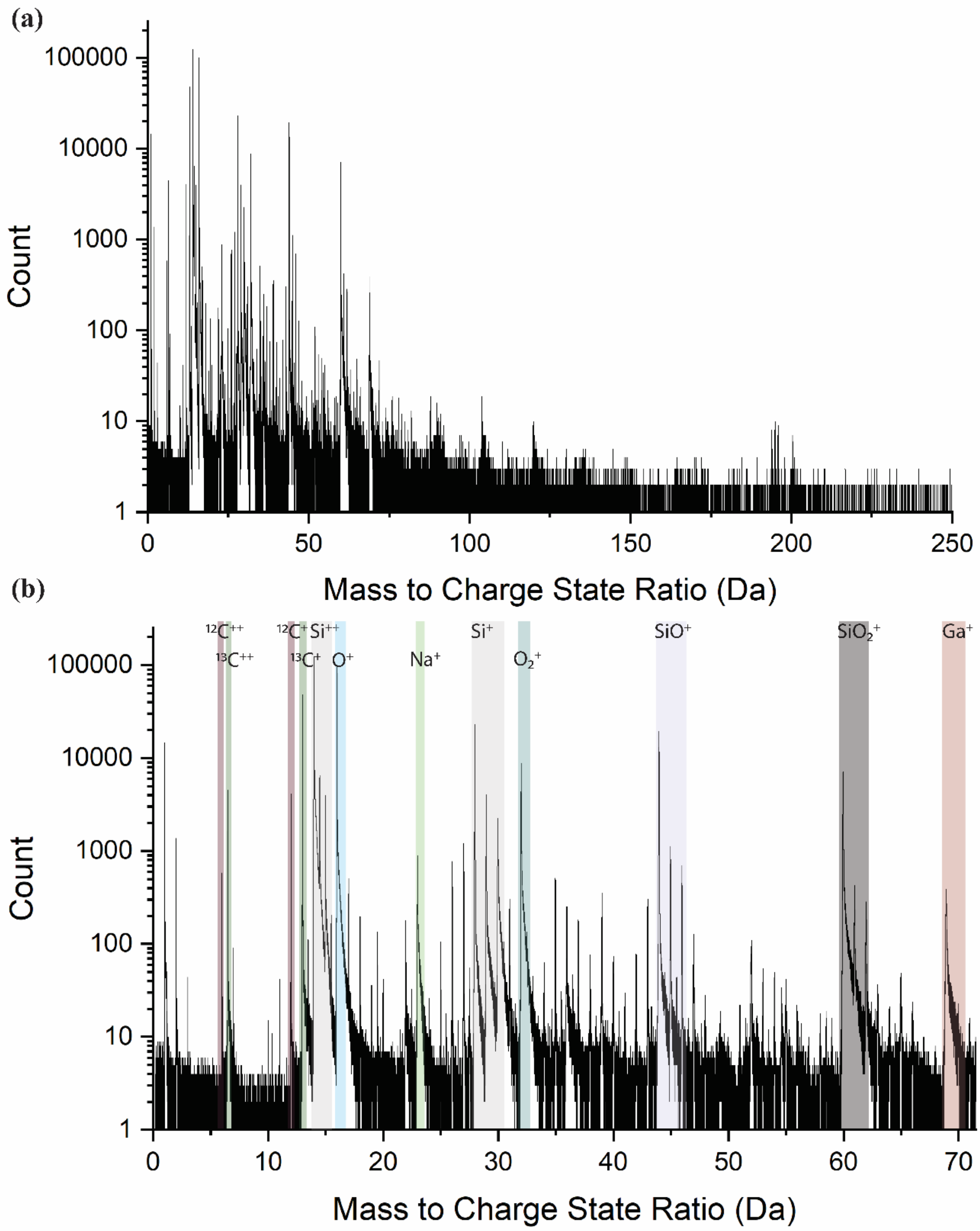

Figure S5: (a) Full mass spectrum obtained from the APT analysis reconstructed in Figure 4. (b) Lower mass-to-charge-state-ratio range of the same mass spectrum, highlighting the identity of the major peaks.

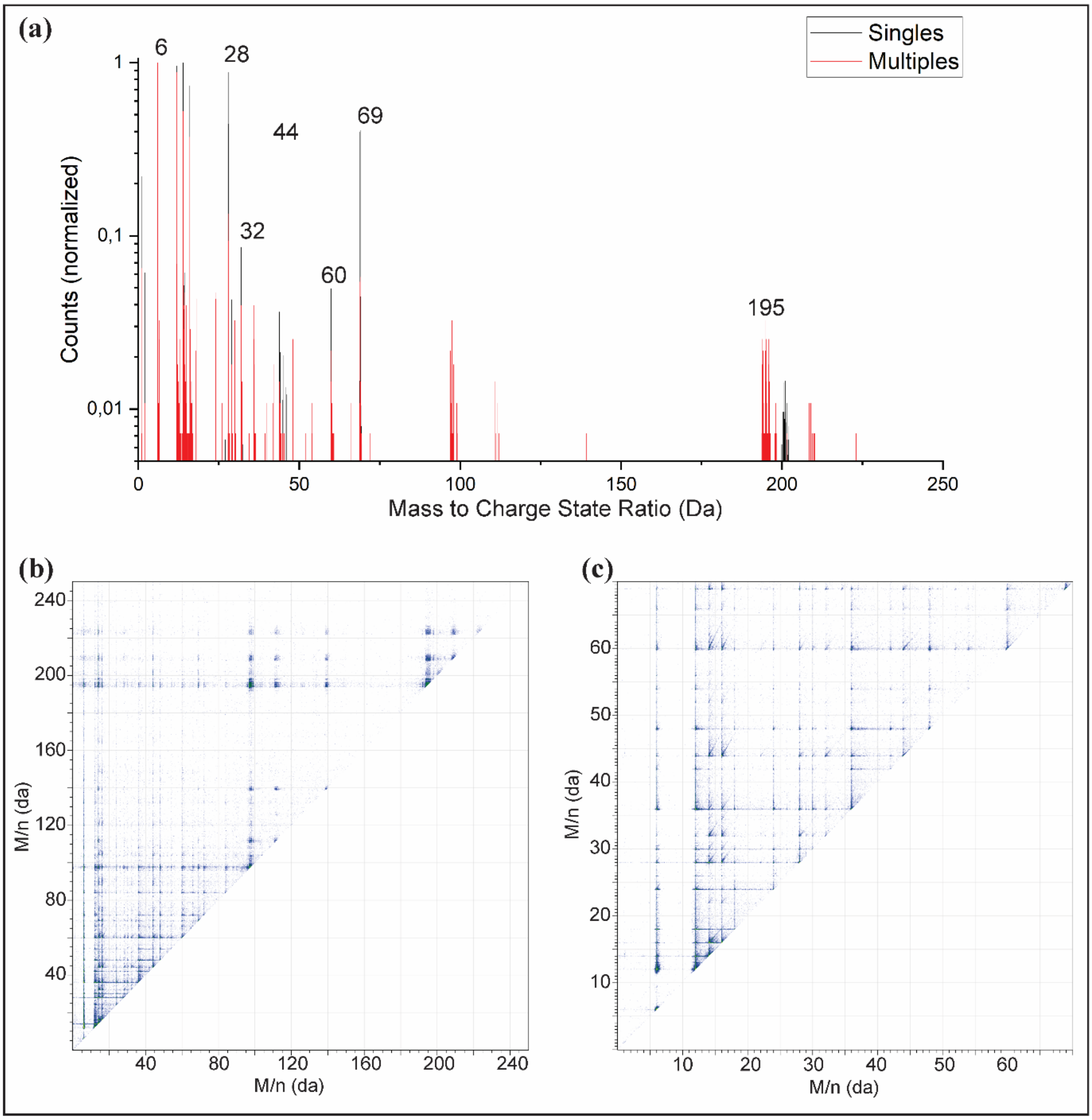

Figure S6: (a) Normalized mass spectra of the single and multiple events obtained from the APT analysis in Figure 4. The analysis was carried out with an LPE of 250 pJ. (b) Full correlated evaporation histogram from the same analysis. (c) Lower mass-to-charge-state-ratio excerpt of the histogram in (b).

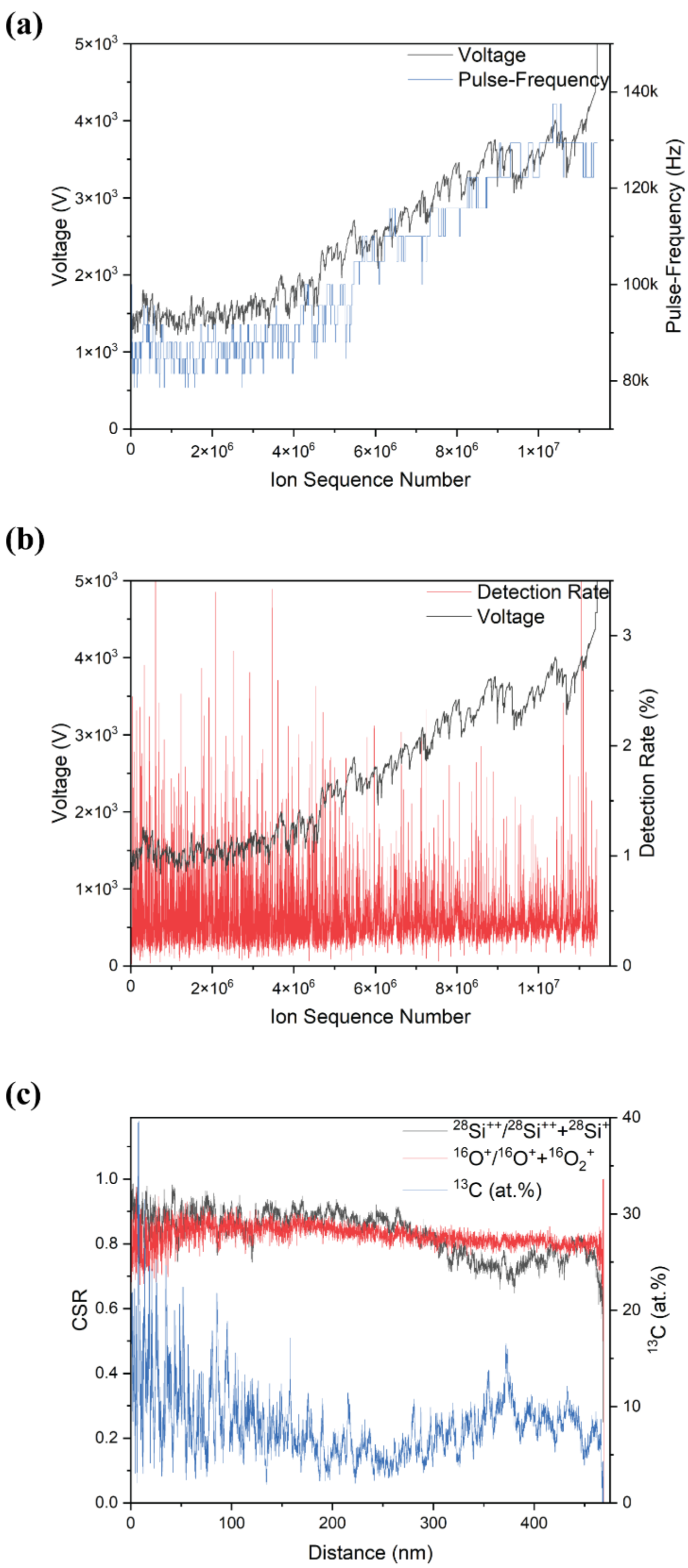

Figure S7: (a) Voltage and pulse frequency history from the APT analysis in Figure 4. (b) Voltage and detection rate history from the same analysis. (c) CSR of silicon and oxygen together with the 13C atomic concentration from a 1D concentration profile along the z-direction of the same analysis.

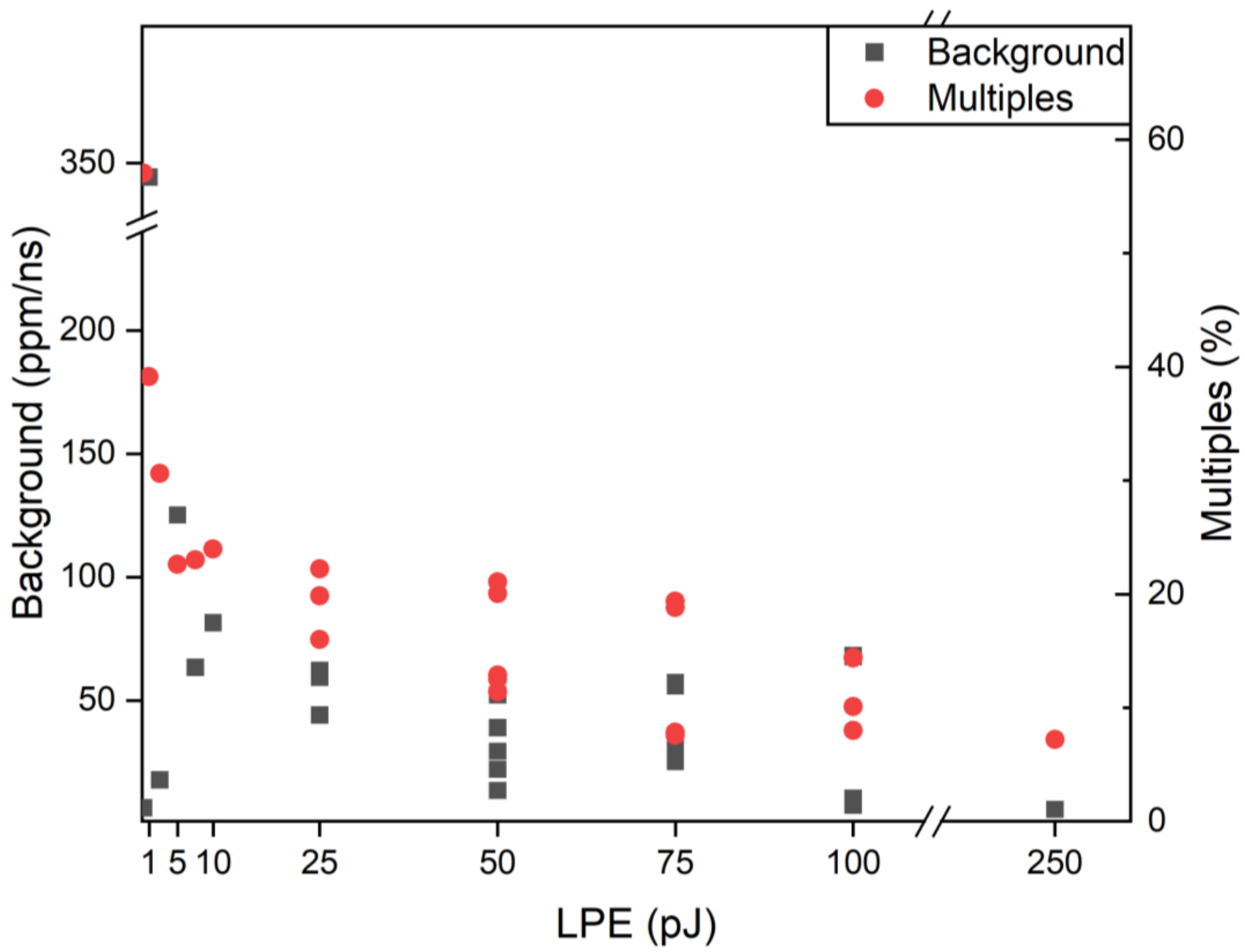

Figure S8: Background and fraction of multiple events for the corresponding data points in Figure 5.

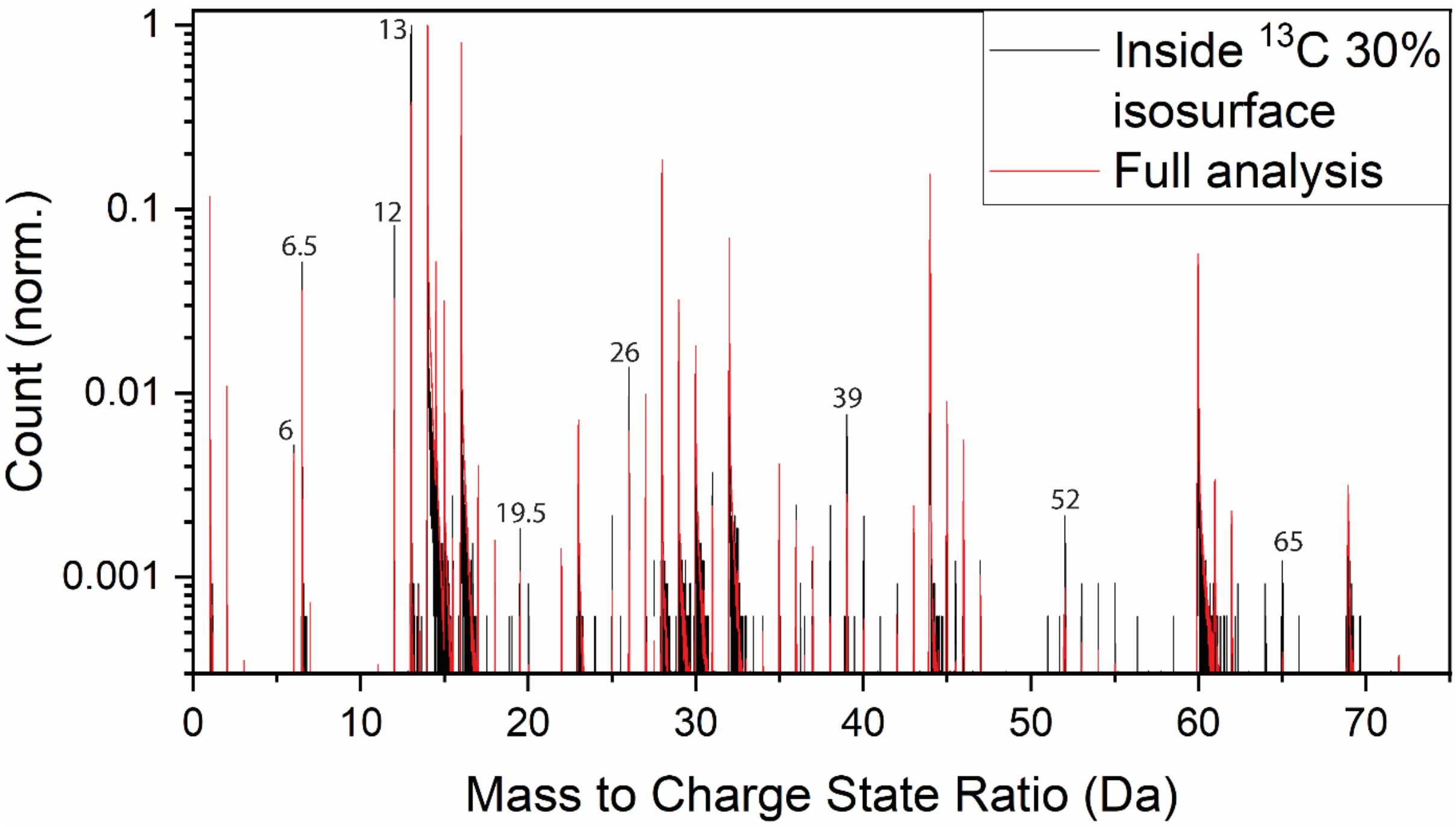

Figure S9: Comparison of normalized mass spectra obtained from the regions defined by the isoconcentration surfaces set to 30 atomic % ^{13}C and the full run from the analysis shown in Figure 4. The peaks found to be relatively higher inside the isoconcentration surface are labelled.

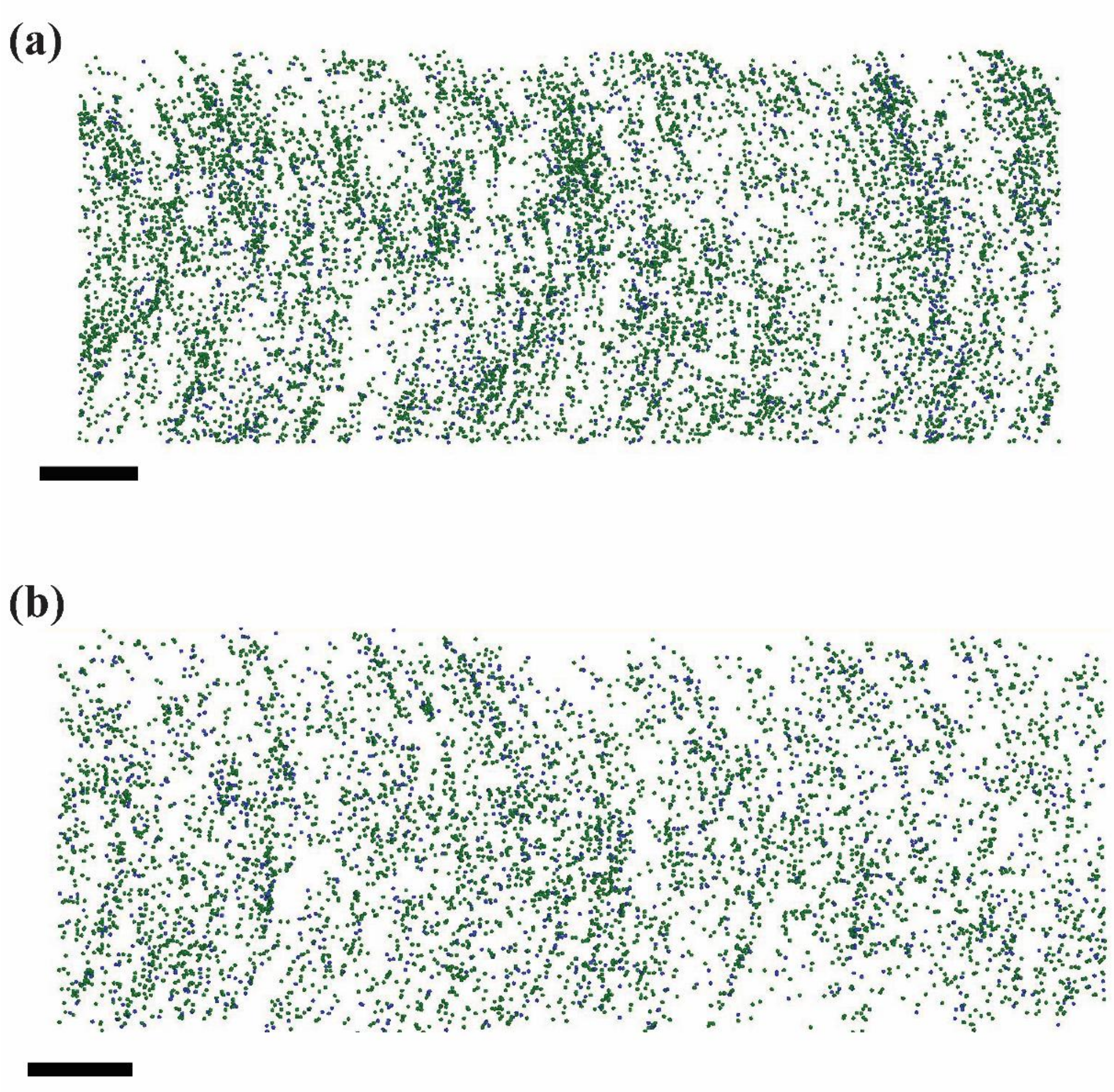

Figure S10: 50x20x10 nm segments of 3D atom maps obtained during APT analysis at LPEs of (a) 50 pJ and (b) 2.5 pJ. $^{13}C^{+}$ and $^{13}C^{++}$ are shown as green and blue spheres respectively. Scale bars: 5 nm.

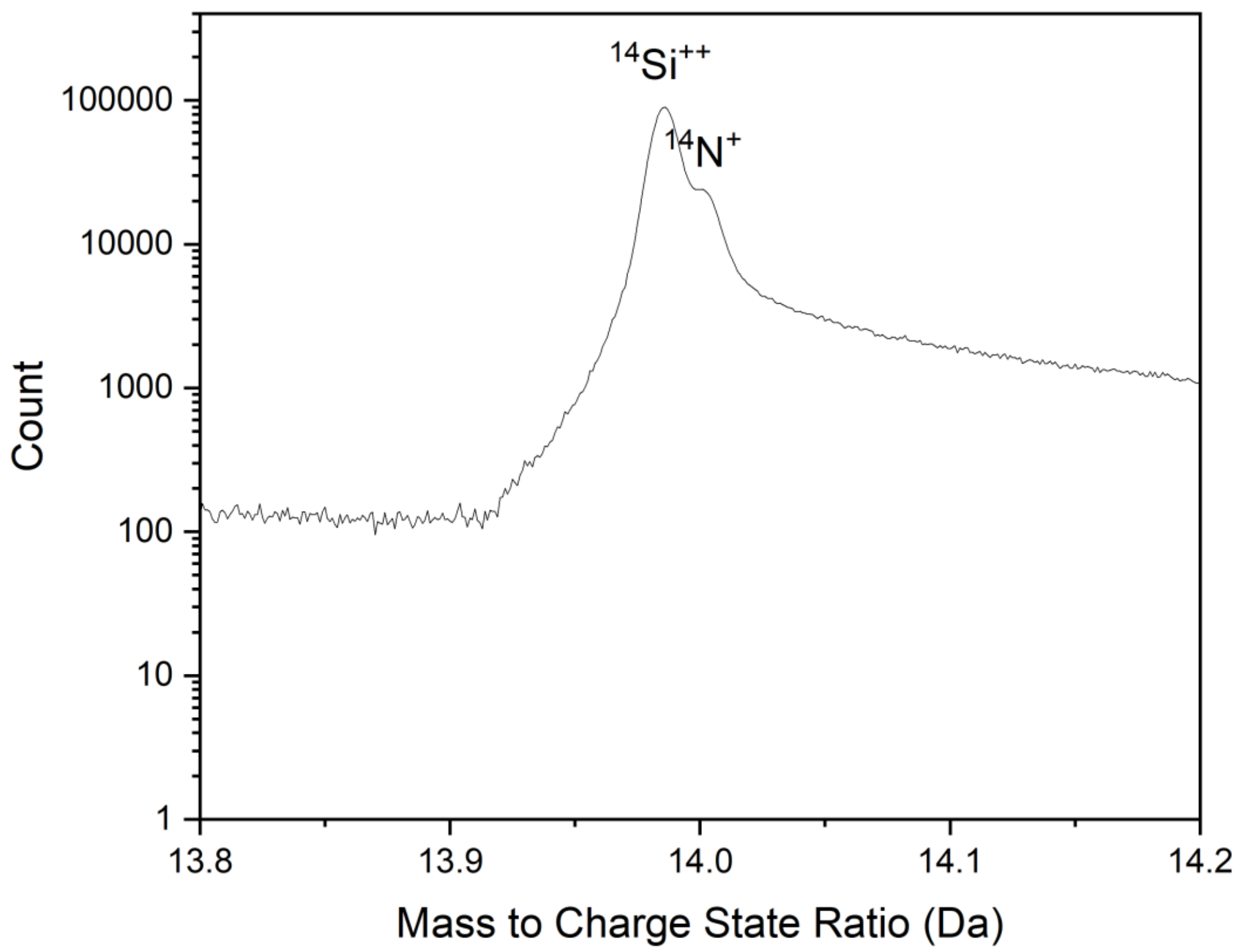

Figure S11: Excerpt of the mass spectrum obtained from the analysis with the highest relative detected amount of ^{13}C. The main $^{14}Si^{++}$ peak is flanked by a shoulder corresponding to the mass to charge state ratio of $^{14}N^{+}$.

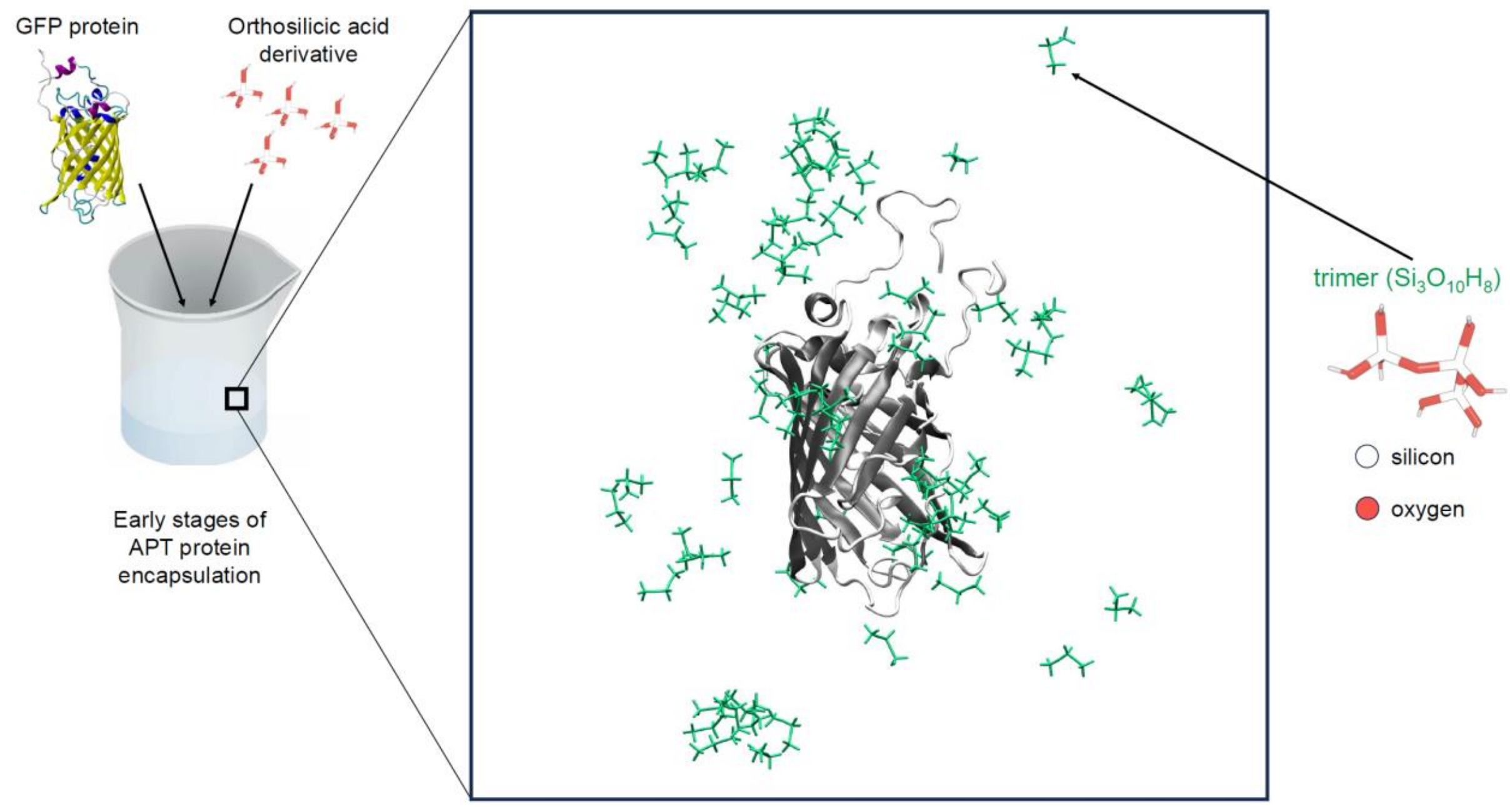

Figure S12: Scheme of the conceptual workflow of the Molecular Dynamics simulations. The starting configuration of a system composed of GFP and silica at 1.2 M concentration is reported in the simulation box on the right, with the protein and orthosilicic acid trimers displayed in grey and green, respectively.

Tables

Table S1: The data presented in graphs Figure 5 and Figure S8, together with the number of detected ^{13}C counts in the analyses.

LPE	Background (ppm/ns)	Multiples (%)	$^{13}C/^{28}Si^{++}$	^{13}C counts ($^{+}$ and $^{++}$)	Counts (total)
**250**	5.872	7.19	0.135	176027	11203948
**100**	67.8	14.393	0.141	12977	306 061
**100**	7.598	7.98	0.219	115785	93 812
**100**	10.37	10.11	0.266	1041515	21 580 277
**100**	68.25	14.39	0.145	42987	1 462 213
**75**	25.28	7.56	0.222	55123	1 427 468
**75**	32.4	7.84	0.2245	13820	611 115
**75**	55.842	18.83	0.2	36958	848 908
**75**	57.2	19.37	0.093	19208	888 875
**50**	13.535	12.53	0.275	15873	710 780
**50**	52.3	21.09	0.187	32526	901 642
**50**	29.4	12.87	0.163	20167	555 978
**50**	38.99	11.39	0.311	8752	61 910
**50**	22.12	20.04	0.119	23434	887 551
**25**	59.3	22.23	0.132	27624	908 321
**25**	62.27	19.85	0.058	35284	2 737 797
**25**	44.108	16.005	0.095	5281	602 650
**10**	81.5	23.97	0.0898	8274	371 454
**7.5**	63.4	23.01	0.078	53403	2 988 819
**5**	125.2	22.61	0.08	14892	945 375
**2.5**	17.8	30.64	0.11	20820	933 268
**1**	344.88	39.13	0.076	5933	570 455
**0.5**	6.448	57.07	0.117	9423	1 015 392
